# 708 Common and 2010 rare *DISC1* locus variants identified in 1542 subjects: analysis for association with psychiatric disorder and cognitive traits

**DOI:** 10.1038/mp.2013.68

**Published:** 2013-06-04

**Authors:** P A Thomson, J S Parla, A F McRae, M Kramer, K Ramakrishnan, J Yao, D C Soares, S McCarthy, S W Morris, L Cardone, S Cass, E Ghiban, W Hennah, K L Evans, D Rebolini, J K Millar, S E Harris, J M Starr, D J MacIntyre, Generation Scotland, A M McIntosh, J D Watson, I J Deary, P M Visscher, D H Blackwood, W R McCombie, D J Porteous

**Affiliations:** 1 Medical Genetics Section, University of Edinburgh Molecular Medicine Centre, MRC Institute of Genetics and Molecular Medicine, Western General Hospital, Edinburgh, UK; 2 Centre for Cognitive Ageing and Cognitive Epidemiology, Edinburgh, UK; 3 Stanley Institute for Cognitive Genomics, Cold Spring Harbor Laboratory, Cold Spring Harbor, NY, USA; 4 University of Queensland Diamantina Institute, The University of Queensland, Princess Alexandra Hospital, Brisbane, QLD, Australia; 5 Institute for Molecular Medicine, Finland FIMM, University of Helsinki, Helsinki, Finland; 6 Division of Psychiatry, University of Edinburgh, Royal Edinburgh Hospital, Edinburgh, UK; 7 Generation Scotland, A Collaboration between the University Medical Schools and NHS, Aberdeen, Dundee, Edinburgh and Glasgow, UK; 8 Queensland Brain Institute, The University of Queensland, Brisbane, QLD, Australia

**Keywords:** *DISC1*, recurrent major depressive disorder, sequencing

## Abstract

A balanced t(1;11) translocation that transects the *Disrupted in schizophrenia 1 (DISC1)* gene shows genome-wide significant linkage for schizophrenia and recurrent major depressive disorder (rMDD) in a single large Scottish family, but genome-wide and exome sequencing-based association studies have not supported a role for *DISC1* in psychiatric illness. To explore *DISC1* in more detail, we sequenced 528 kb of the *DISC1* locus in 653 cases and 889 controls. We report 2718 validated single-nucleotide polymorphisms (SNPs) of which 2010 have a minor allele frequency of <1%. Only 38% of these variants are reported in the 1000 Genomes Project European subset. This suggests that many *DISC1* SNPs remain undiscovered and are essentially private. Rare coding variants identified exclusively in patients were found in likely functional protein domains. Significant region-wide association was observed between rs16856199 and rMDD (*P*=0.026, unadjusted *P*=6.3 × 10^−5^, OR=3.48). This was not replicated in additional recurrent major depression samples (replication *P*=0.11). Combined analysis of both the original and replication set supported the original association (*P*=0.0058, OR=1.46). Evidence for segregation of this variant with disease in families was limited to those of rMDD individuals referred from primary care. Burden analysis for coding and non-coding variants gave nominal associations with diagnosis and measures of mood and cognition. Together, these observations are likely to generalise to other candidate genes for major mental illness and may thus provide guidelines for the design of future studies.

## Introduction

Schizophrenia (SZ), bipolar disorder (BD) and recurrent major depressive disorder (rMDD) are common forms of serious mental illness, each with a strong and overlapping genetic component.^1, 2, 3^ Genome-wide linkage, association, cytogenetic, copy number variant and, more recently, sequencing studies establish that the genetic architecture of psychiatric illness is complex and that there is extensive genetic heterogeneity, which is incompletely defined or understood (reviewed in Sullivan *et al.*^4^). We previously reported a t(1;11) translocation in a single large Scottish family that showed genome-wide significant linkage for SZ, rMDD and jointly with BD.^5^ The t(1;11) translocation is balanced and structurally simple, but the outcome is genetically complex, disrupting the protein coding gene *Disrupted in schizophrenia 1 (DISC1),* the antisense non-coding gene *Disrupted in schizophrenia 2 (DISC2)* and the non-coding gene DISC1FP1, creating a DISC1/*DISC1FP1* fusion transcript.^6, 7, 8, 9, 10, 11^

Several small independent studies have reported evidence for association of single *DISC1* single-nucleotide polymorphisms (SNPs) (coding and non-coding) or haplotypes with SZ, BD, rMDD and other neuropsychiatric traits, including autism spectrum disorder, cognition, normative cognitive ageing, anxiety and structural and functional brain imaging phenotypes.^9, 12, 13^ Rare amino-acid substitution variants in DISC1 have been reported in cases of SZ,^10, 11^ BD,^14^ rMDD,^13^ autism spectrum disorder^15^ and agenesis of the corpus callosum,^16^ as has an increased burden of rare missense variants in exon 11 of *DISC1* for schizoaffective disorder,^17^ and for DISC1 pathway genes in SZ.^10^ In contrast, a meta-analysis of all known common variants within the DISC locus, from a total of 11 626 cases and 15 237 controls that involved the testing of 1241 SNPs, found no evidence that common variants at the DISC locus are significantly associated with SZ.^18^ Moreover, the *DISC1* locus has not reached genome-wide significance in large-scale meta-analyses of linkage studies of SZ,^19^ nor have its common variants in large-scale genome-wide association studies of SZ, BD or rMDD.^20, 21, 22^ A recent exon-based study that sequenced 2.7 kb of *DISC1* in 727 cases of SZ and 733 controls found 32 rare alleles (minor allele frequency (MAF)<0.01) in SZ cases and 40 in European controls with no evidence for a significantly increased burden of likely pathogenic variants.^23^ *DISC1*, however, continues to feature strongly in attempts to assess genome-wide association results in terms of networks^24^ or in combination with known biological function.^25, 26, 27^

The biological functions of *DISC1* fit well with current aetiological concepts in SZ-related major mental illness and cognition.^28^ DISC1 is a scaffold protein that interacts with, and modulates the activity of, multiple proteins with key roles in neurodevelopment, neurogenesis, neuronal migration, integration and signalling^29, 30, 31^ including the antidepressant and antipsychotic targets GSK3β^32^ and PDE4.^33^ Several common and rare amino-acid polymorphisms of *DISC1* have predicted deleterious effects on protein function and demonstrable biological effects in experimental settings.^31^ The 704C allele is associated with reduced activity of ERK1 and Akt kinases, altered binding affinities of DISC1 for NDE1 and NDEL1 and variation in DISC1 oligomeric status.^34, 35, 36, 37^ The 607F allele results in (a) reduced binding and centrosomal localisation of PCM1, (b) reduced noradrenaline neurotransmitter release in SH-SY5Y cells,^38^ (c) altered mitochondrial trafficking^39^ and (d) a partial shift from neuronal to glial expression in the brain.^40^ Furthermore, Singh *et al.*^41^ reported that 607F impacts negatively on neural progenitor proliferation in E16 mouse brain, correlates with aberrant *wnt* signalling in human lymphoblasts, and is associated with a neurodevelopmental phenotype in morpholino mutant zebrafish. R37W lies within an arginine-rich nuclear localisation motif and a partially overlapping interaction domain for PDE4^42^ and GSK3β.^41, 43^ The 37W allele shows reduced nuclear DISC1 expression and altered DISC1 regulation of ATF4, a critical modulator of cAMP signalling and mediator of the stress response.^44^

In summary, whereas the primary genetic evidence from the original Scottish t(1;11) family was significant at the genome-wide significance level for both SZ and rMDD and the experimental evidence and biological plausibility of DISC1 remains strong, the evidence from subsequent linkage and candidate gene association studies is however inconsistent and not supported by genome-wide association studies or meta-analysis.^12^ To explore these contrasting findings, we aimed here to establish the nature and frequency of *DISC1* genomic sequence variants, identify rare variants in putative functional domains, and test for effects of these on cognitive traits and the risk of psychiatric illness. We comprehensively sequenced 528 kb covering the entire *DISC1* locus, including *TRAX* (also known as *TSNAX*) for which there is evidence for intergenic splicing with DISC1^45^ and the intergenic region, which contains regulatory elements immediately 5’ of *DISC1.*^13, 46, 47, 48^

## Materials and methods

A full summary of the methods can be found in Supplementary Information. Briefly, all study participants gave signed consent for their data and samples to be used in studies that have been approved by the appropriate Research Ethics Committee or the GS access Committee. Genomic DNA from each individual was whole genome amplified in triplicate, the products pooled and amplified with primer pairs tiled across 528 kb of *TRAX/DISC1* (hg18 chr1:229723339-230251606; hg19 chr1:231656716-232184983). For each sample, the pooled products were sheared, converted into paired-end Illumina libraries and sequenced on an Illumina GAII or HiSeq 2000 sequencer to >80% coverage and >30-fold depth. Sequences were aligned to the UCSC hg18 reference sequence, variants called using MAQ software^49^ and the variants in repeats removed. Ten percent of all remaining variants were validated using Sanger sequencing chemistry on an ABI3730 sequencer, and the derived information used to optimise the quality control filters. After quality control screening, all exonic and low frequency (MAF<1%) variants were also validated by Sanger chemistry sequencing as above. The variants were functionally annotated using SNPnexus^50^ (http://www.snp-nexus.org). Non-coding variants were annotated using the UCSC table browser for the following tracks: ‘RepeatMasker’,^51^ ‘CpG island’, ‘TFBS conserved’, ‘7x Reg Potential’ (which substantially overlaps with DNAse hypersensitivity sites) and/or ‘28-Way Most conserved—PlacMammal’ (http://genome.ucsc.edu/). Sequence variants classified as coding were mapped to the DISC1 L isoform and potential pathogenicity ascribed using Pmut,^52^ Panther^53^ and PolyPhen-2.^54^ The coding sequence variants were mapped onto a list of known curated DISC1-interactor binding sites^31^ and with other functional elements (for example, phosphorylation sites^55^). Case–control association was tested on the combined case samples as well as individually for SZ, BP and rMDD using Fisher’s exact test. Permutation was used to derive region-wide *P*-values and significance thresholds. Quantitative trait association analyses using LBC1936 samples were performed by linear regression of the trait residuals (adjusted for age and sex) on the number of minor alleles at each SNP, with empirical *P*-values estimated by permutation to avoid issues with the test statistic distribution caused by the combination of rare variants and slight deviations from normality in the phenotypes. All association analyses were performed using PLINK.^56^ Mark-recapture analysis followed the Lincoln-Petersen and Modified Petersen methods^57, 58^ with 95% confidence intervals calculated following Chapman.^59^ Burden analysis was performed in PLINK/SEQ to implement BURDEN and VTTEST with empirical *P*-values estimated using permutation. Genotyping of the replication and familial samples was performed by the Edinburgh Wellcome Trust Clinical Research Facility Genetics Core using TaqMan SNP genotyping assay C__33950433_10 with concurrent genotyping of known heterozygotes.

### Data access

The accession numbers for sequence data are NCBI ss472328925—ss472331023.

## Results

### Sequence analysis

We sequenced 1542 Caucasians from Scotland comprising 240 cases of SZ, 221 cases of BD, 192 cases of rMDD and, as controls, 889 members of the Lothian Birth Cohort 1936 (LBC1936), which have been extensively phenotyped.^60^ Each sample was sequenced to >80% coverage at a minimum of 30-fold read depth by long-range PCR and sequencing on either Illumina GAII or HiSeq 2000 sequencers. To ensure a robust data set, all variants within repetitive regions were removed. Final quality score thresholds for the data were derived from capillary sequence validation of 10% of the remaining variants. All variants with an MAF <1% were validated by ABI3730 sequencing. After quality control, there was no evidence for sequencing bias between cases and controls (Supplementary Figure S1). Allele frequencies from our sample showed strong concordance to those from the European subset of the 1000 Genomes Project^61^ (Supplementary Figure S2). We report 2718 SNPs in the 1542 samples analysed, 708 at ⩾1% and 2010 at <1% MAF (Supplementary Table S1). Only 1027 of the 2718 SNPs (38%) were previously reported in the European subset of the 1000 Genomes Project.^61^

**Figure 1:**
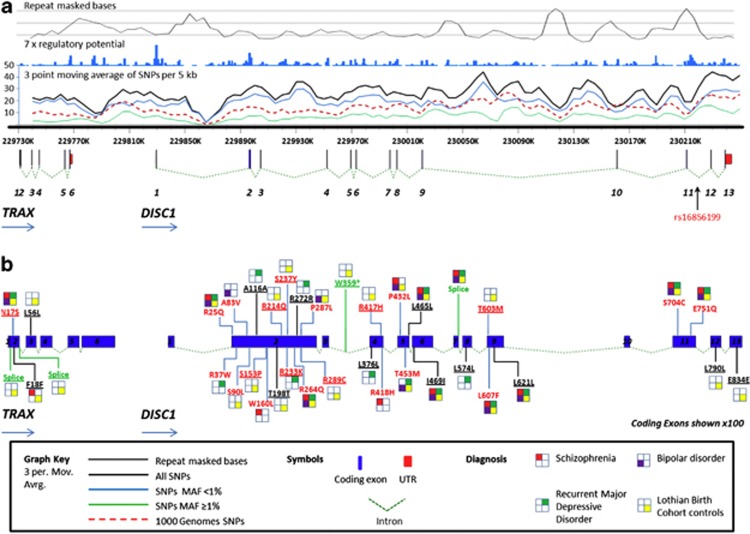
TRAX/DISC1 (*Disrupted in schizophrenia 1*) genomic and exon structure: alignment of coding and regulatory variants. (**a**) Three-period moving average of all single-nucleotide polymorphisms (SNPs) identified per 5 kb across the region in this study with TRAX/DISC1 intron/exons structure given to scale. Total SNP number (black), those with a minor allele frequency (MAF) of <1% SNPs (blue), those ⩾1% MAF (green), rs16856199 (arrow). For comparison, the number of SNPs identified in the 1000 genomes (red, dashed) and the number of bases repeat masked (top black) and 7x regulatory potential (top blue) are also shown. Exon and intron structure of TRAX and DISC1 are drawn to scale. (**b**) The position and diagnoses of exonic or regulatory SNPs. SNPs not seen previously (underlined), synonymous SNPs (black) and non-synonymous SNPs (red), stop or putative splice SNPs (green). Novel SNPs not previously reported in the European samples of the 1000 Genomes Project (v3.20101123) or the NHLBI GO Exome Sequencing Project (ESP6500) or relevant sequencing and association studies^10, 16, 17, 64, 65^ are underlined.

As defined and annotated by the UCSC genome browser (http://genome.ucsc.edu), 489 SNPs mapped to regions of regulatory potential, 177 to non-coding exons (including DISC2) and 36 to coding regions of exons. Of these 36 variants, 12 were synonymous changes, 23 were non-synonymous changes, with one producing a stop codon consistent with the DISC1 Es isoform (Figure 1; Supplementary Figure S3; Supplementary Tables S1 and S2). Supplementary Table S3 summarises the overlap between variants identified in this study and other DISC1 sequencing studies and relevant association studies.^10, 11, 13, 14, 16, 17, 23, 46^

### Association and segregation of common variants with psychiatric illness and related quantitative traits

**Figure 2:**
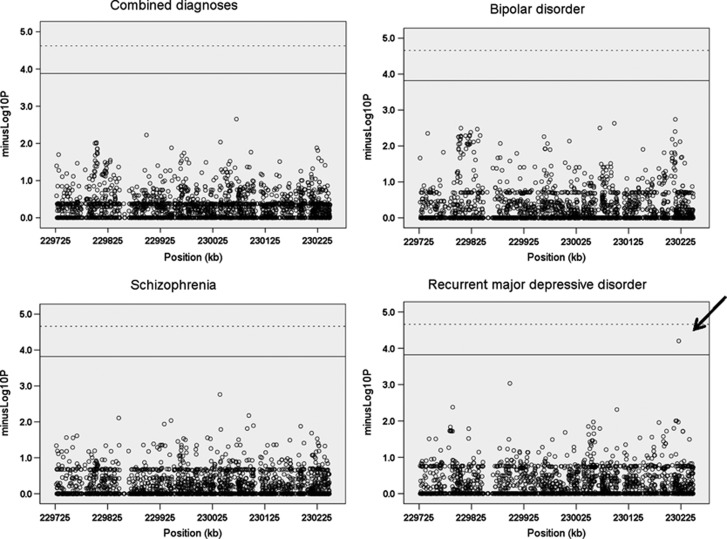
Region-wide association analysis for schizophrenia, bipolar and recurrent major depressive disorder. Nominal *P*-values for Fisher’s exact tests are plotted against genomic location (hg18) across the *TRAX/DISC1 (Disrupted in schizophrenia 1)* locus. Reference lines represent 1% (dashed) and 5% (solid) region-wide empirical thresholds. Only the association of rs16856199 and recurrent major depressive disorder remains significant at the 5% threshold (arrow).

Genome-wide association studies of SZ, BP and rMDD are most consistent with a polygenic liability for common variants, but they also imply that there is real ‘missing’ genetic variation, which is most likely due to risk variants having low frequency in the population. To test for evidence of *DISC1* association, we applied the Fisher’s exact test across all variants and all diagnoses (Figure 2). There was no evidence for SNP association at genome-wide levels of significance for any diagnosis when considered separately or combined, nor was there evidence for locus-wide association of variants with SZ or BP. We did detect a novel, locus-wide empirical association *P=*0.026 (OR=3.48, 95% CI=1.95–6.23, unadjusted *P*=6.3 × 10^−5^) between intronic variant rs16856199 and rMDD. We speculated that individual risk alleles might be predicted to segregate with disease in families. Twelve additional family members were available for genotyping for four rs16856199 carriers. The rs16856199 risk allele segregated with rMDD in all four families (Supplementary Figure S4).

We next tested for association of rs1685199 with depression in three additional sample sets: a group of individuals referred from primary care to a hospital outpatient clinic (*n*=467 rMDD patients), and two population-based samples drawn from primary care as part of the Generation Scotland: Scottish Family Health Study consisting of 645 cases with rMDD and 690 cases with single episode MDD. All three groups were compared with 4017 controls drawn exclusively from Generation Scotland: Scottish Family Health Study (Supplementary additional text and Table S4). No significant association was seen with any individual replication set or all three combined (best *P*=0.088). Analysis of all three rMDD sets, both the original set and the two rMDD replication sets, was supportive of association (1112 rMDD, 4017 controls; *P*=0.0058, OR=1.46, 95%CI=1.12–1.91). Combined analysis of both sets of individuals referred from primary care showed stronger nominal association (*P*=0.00065, OR=1.76, 95%CI=1.27–2.44). No association was seen in the combined analysis of both the rMDD and MDD population-based replication sets (*P*=0.41). The risk allele for rs16856199 did not segregate with rMDD in 10 families of carriers identified from the Generation Scotland replication sample (Supplementary Figure S5). This suggests that there is increased evidence for association of rs16856199 in the more severely affected individuals.

SNP rs16856199 is on the Affymetrix 6.0 array, but the best tagging SNP on the Illumina 660W-Quad, Human Hap, Human1M-Duo arrays is rs16856189. SNP rs16856189 has an *r*^2^ of 0.27 with rs16856199, which may explain in part why this association has not been reported previously in genome-wide association studies.^22, 62, 63^ SNP rs6678723, which lies 2.1 kb distal to this SNP within intron 11, showed the most significant association of *DISC1* in the recent mega-analysis of depression (*P*=0.0092).^20^

The LBC1936 has quantitative measures of symptoms of anxiety, depression and the personality trait of neuroticism, plus psychometrically tested measures of cognitive ability (fluid (age sensitive) and crystallised (non-age sensitive)) and cognitive ageing,^60, 64^ which have been shown to be highly heritable and polygenic.^65, 66^ Association of these traits with *DISC1* was tested by linear regression analyses, co-varied for age at testing and sex (Supplementary Figures S6–S8). There were no region-wide significant findings for any of these quantitative traits.

### Estimating the net pool of *DISC1* variants

To estimate the effective pool of common and rare sequence variants in the European population, we applied a ‘mark-recapture’ approach (see Supplementary methods) to our data and that of the 1000 Genomes Project (v3.20101123)^67^ after appropriate checks on read depth and Sanger sequence validation (Supplementary Table S5; Supplementary Figure 9). The total number of *DISC1* SNPs ⩾1% MAF was estimated at 905 (95%CI=905±5), of which 901 (99.5%) are known (Supplementary Table S6). The number of rare variants (<1% MAF) is less confidently predicted, but is likely to be substantially higher (95%CI=3777±252) (Supplementary Table S6). Thus, despite the ∼2500 European genomes in which the *DISC1* locus has been completely sequenced and the 2305 rare *DISC1* variants now known, ∼40% or more remain to be discovered, and will be essentially ‘private’.

### Rare amino-acid substitutions

Of the 17 rare coding SNPs previously reported for *DISC1*,^10, 16, 17, 68, 69^ we identified 12 (70.6%) plus an additional 8 non-synonymous variants, of which 5 are also absent from the European samples of both the 1000 Genomes Project (v3.20101123)^61^ and the Exome Variant Server (NHLBI GO Exome Sequencing Project (ESP), Seattle, WA; http://evs.gs.washington.edu/EVS/; September, 2012), and previous *DISC1* sequencing studies.^10, 11, 13, 14, 16, 17, 23, 46, 70^ (Figure 1; Supplementary Figure S3; Supplementary Tables S2 and S3).

Five variants, R37W, T453M, T603M, L607F and S704C, are predicted to be deleterious by all three applied prediction algorithms (PolyPhen-2, Pmut and Panther; Supplementary Table S2). From this set, the functional effects of the common variants L607F (rs6675281) and S704C (rs821616) have been well documented in the literature, as mentioned earlier. R37W lies within a defined nuclear localisation signal^71^ and PDE4B binding site^72^ and is seen in a single case of rMDD (discussed at the end of this section). T453M is present at low frequency in cases and control individuals, both in this study and others (Supplementary Table S3). T603M was only identified in a single control, but Song *et al.*^11^ reported a T603I variant in a schizophrenic individual that was absent in their set of control individuals (Supplementary Table S3).

Five variants were only observed in affected individuals from our study (R37W, A83V, W160L, R233K and R418H), but not in 889 control individuals. Four of these non-synonymous case-only singletons are located in the largest coding exon, *DISC1* exon 2, and the remaining variant is in *DISC1* exon 4 (Figure 1). A83V was seen in a single individual with BD; this variant is predicted to be deleterious by PolyPhen-2 and Pmut and has been shown to affect wnt signalling.^41^ It was however observed at low frequency in controls and in individuals with partial agenesis of the corpus callosum in previous studies (Supplementary Table S3).^11, 14, 16^ Apart from R37W and A83V, none of the three remaining non-synonymous variants, W160L, R233K and R418H, are consistently predicted by the three prediction algorithms to have functional effects (Supplementary Table S2). 160L and 418H were detected in single SCZ individuals and have also previously been reported in individuals with SCZ; but 160L has also been detected in control individuals.^10^ The variant 233K has not been previously reported, and was identified in an individual with rMDD. No non-synonymous variants in *TRAX* were found in cases only. A single stop mutation was identified in a control individual and produces an alternative stop site for the DISC1 Es isoform. These variants can now be tested for potential impact on DISC1 biophysical properties, protein interaction and biological function.^29, 30, 31^

**Figure 3:**
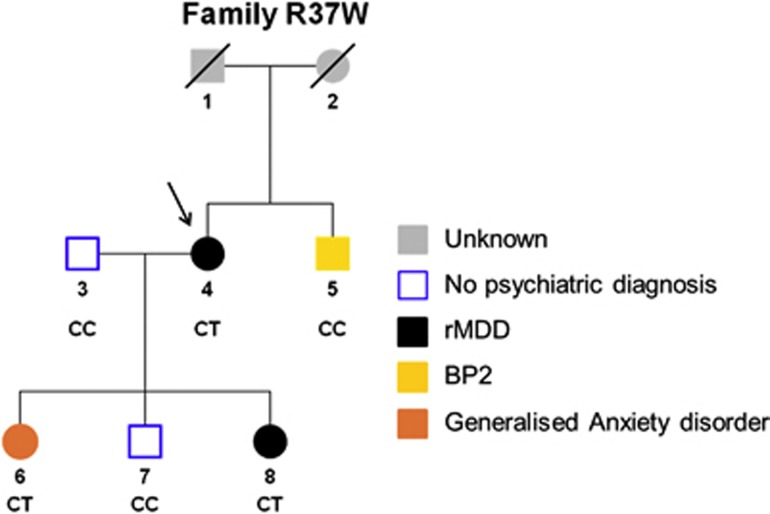
Segregation of the R37W polymorphism with psychiatric diagnoses in a small Scottish family. The proband of the family is indicated (arrow). The codon containing the T allele encodes for the amino acid tryptophan (W) and the codon containing the A allele encodes arginine (R). rMDD, recurrent major depressive disorder; BP2, bipolar II.

Evidence for familial segregation was sought for five rare exonic variants where additional family members were available, but none segregated perfectly or unequivocally with diagnosis (Supplementary Figure 9). Of note however was the identification of the non-synonymous amino-acid variant R37W (rs137948488), first reported^68^ in a subject with SZ, and seen here in a single case of rMDD. R37 is strictly conserved among orthologues and recent publications, including our own, have demonstrated biological effects of 37W on DISC1 interactions,^32, 73^ and shown a dominant-negative effect on the sub-cellular distribution of DISC1.^44^ Five additional family members of the 37W carrier were available for genotyping diagnosed with rMMD, generalised anxiety disorder, bipolar II or no psychiatric diagnosis at the time of assessment. The R37W mutation was present in relatives with rMDD and generalised anxiety disorder, but not in a relative with bipolar II, or any unaffected individual (Figure 3).

### Burden analysis for putative functional variants

To explore the burden of SNPs of potential functional significance, all variants with MAF <1% were first validated by ABI3730 sequencing. There was no significant overall difference in the number of singleton variants (Supplementary Figure S10) or in the overall number of minor alleles by diagnosis (see Supplementary Methods). SNPs were classified on the basis of bioinformatic annotation into seven functional classes: those in exons including untranslated exons, coding sequence, non-synonymous coding SNPs, conserved regions, regions with regulatory potential, conserved transcription factor binding sites and CpG islands (see Materials and methods and Supplementary Table S7). The empirical *P*-values for the burden analysis were obtained by permutation correcting for the multiple thresholds tested, but not for the multiple functional subgroups or diagnostic classes (SZ, BP and rMDD and all cases combined), therefore all results are reported as nominal significance values (Supplementary Table S8; Supplementary Methods). Details of nominally significant results are given in Table 1. For rMDD only, there was a nominally significant (*P*=0.044) excess of minor alleles for SNPs with regulatory potential across all frequencies, and for rare SNPs in conserved regions with MAF ⩽0.18% (*P*=0.022). Nominally significant association was found in the LBC1936 data between the burden of minor alleles across all frequencies for SNPs in conserved transcription factor binding sites and increased symptoms of depression, a measure of depressed mood at the time of testing (Table 1). In addition, a nominally significant increase in burden of minor alleles for SNPs in CpG islands or coding SNPs was observed with Moray House Test scores, measures of cognitive ability (Supplementary Table S9). Summaries of the nominally significant results are given in Table 1.

## Discussion

Diagnoses of SZ, BP and rMDD were all present in the original Scottish family carrying the translocation that disrupts *DISC1*. All three DSMIV diagnoses have a strong and overlapping genetic component, but robust statistical analysis of gene-level contributions to risk are complicated by extensive genetic heterogeneity within and between diagnoses.^4^ We have provided the most comprehensive landscape of genetic variation at the *DISC1* locus to date in patients with this spectrum of psychiatric illness and in healthy population controls with quantitative measures of mood and cognition. Comparison between our sequencing study and that of the 1000 Genomes Project confirms that current genome-wide association studies effectively captures the majority of common (but not rare) variants in the European population. Our sample size is large by current sequencing study standards, but we lack power to detect genome-wide significant *P*-values for either common or rare variants (see Supplementary Information and Supplementary Figure S11 for further details and also Kiezun *et al.*).^74^ Indeed, the predicted abundance of independent rare variants at this (and any other given) locus makes it highly improbable that any one will contribute to illness in the population at a frequency that will be statistically significant, given the numbers of patients we can afford to analyse by direct sequencing.^74^

We observed no evidence for association at the whole-genome level of statistical significance between individual rare or common variants and either psychiatric illness or cognition. This is consistent with recent findings,^74^ which suggest that much larger samples would be required to detect such associations. Burden analysis of multiple rare and/or deleterious putative functional variants also failed to show association with these traits. We do report both functional and putative regulatory variants that are both individually, and by functional classification, nominally associated with rMDD and/or cognitive ability at the locus-wide level of significance.

Our study identified a novel association between intronic SNP rs16856199 and rMDD in hospital-referral subjects. Segregation with diagnosis in the relatives of these probands corroborated the association, but further studies are required to understand the lack of replication in population-based cohorts with depression. This may be due to inherent differences between patients recruited from hospital-referral compared with those from population-based cohorts. Cohorts from primary care are more likely to have a family history of depression,^75^ and may have more physical and psychiatric comorbidity in general. Conversely, the population-based sample may have shorter, less severe episodes^76^ than the hospital-based cohorts.^77^ However, given the modestly significant *P-*value for rMDD in the discovery cohort, the number of psychiatric traits examined and the lack of replication, it is possible that the observed association is due to chance.

The nominal associations of the burden of common (threshold MAF=30.7%) and rare potentially regulatory variants (threshold MAF=0.060%) to measures of cognitive ability merit further study. A yet-to-be-defined subset of these is likely to have critical roles is spatial and temporal regulation of transcription and splicing.^46, 78^ This highlights the need for annotation tools with improved predictive value for non-coding variants.^79^

More importantly, our study demonstrates that substantial coding and non-coding genetic variation at the *DISC1* locus remains undiscovered. Despite sequencing over 1500 subjects, we have probably captured only ∼40% of the extant *DISC1* variants in just the European population. Crowley *et al.*^23^ sequenced 2.7 kb of *DISC1* exons and 5’ and 3’ regulatory sequence in 1460 samples of European or African origin. We observed 13/38 (34%) of the variants genotyped in the replication phase, supporting the argument for an abundance of rare variants.

The level of sequence variation identified in our study is unlikely to be exceptional, and indeed is consistent with evidence emerging from other genome sequencing studies.^74^ Consequently, it will be challenging to demonstrate robust (replicated) association by statistical evidence alone in case–control studies, exceptionally so with the numbers of patients that are currently affordable for sequencing. The original t(1;11) family illustrates the added issue of variable penetrance and cross-boundary diagnosis for a given mutation: ∼70% of carriers had SZ, BP or rMDD, but ∼30% had no formal psychiatric diagnosis, yet t(1;11) carriers, including both affected and unaffected, had ERP P300 measures in the range typical of individuals with SZ.^5^ The original identification of 37W in a case of SZ and here in a case of rMDD (and two offspring, one with rMDD, the other generalised anxiety disorder) may suggest variable penetrance of this biologically functional variant.^44^ Of note, the 37W variant was not observed in 10 000 control individuals,^11^ the 1000 Genomes project,^61^ the NHLBI GO Exome Sequencing Project (ESP), nor any of our 889 control samples. These findings on R37W reinforce the probable importance of this domain for DISC1 subcellular distribution and binding of interacting proteins^31, 44^ and add to the weight of evidence for other functional studies of DISC1 amino-acid substitutions.^41^ Each observed amino-acid substitution provides a similar opportunity to tease out the relationships between genotype and phenotype and between structure and function.^29, 31^ Overall, these results demonstrate a high level of sequence variation in DISC1, a subset of which may contribute to psychiatric disorder in some individuals who will be typically rare in the population precluding classical statistical analysis and requiring biological validation. This predicts a population-specific contribution of rare casual variants to risk.^80^ Our results indicate the potential value of sequencing non-coding regions of the genome, which may harbour disease-associated regulatory variants. Our findings of both functional and putative regulatory variants nominally associated with depression and cognitive ability merit replication in independent samples and biological exploration.

## Figures and Tables

**Table 1:** Summary of nominally significant burden results

*Case–control*	*Trait*	*SNP subset*	*Test*	*Average excess of variants in cases*	*Optimised MAF threshold (N. SNP)*	P*-value*
	rMDD	RegPot	BURDEN	1.39	—	0.044
	rMDD	PhastCon	VTTEST	1.20	0.0018 (31)	0.022
						
*Quantitative traits*	*Trait*	*SNP subset*	*Test*	*Effect size (beta)*	*Optimised MAF threshold (N. SNP)*	P-*value*
	Symptoms of depression^a^	TFBS	BURDEN	0.062	—	0.032
	Moray House Test at age 70^b^	PhastCon	VTTEST	−0.074	0.0013 (21)	0.030
	Moray House Test at age 70 adjusted for the Moray House Test score at age 11^b^	CpG	VTTEST	−0.064	0.00060 (2)	0.047
	Moray House Test at age 11^b^	Coding	VTTEST	−0.101	0.00070 (12)	0.028
	Moray House Test at age 11^b^	CpG	VTTEST	−0.097	0.31(3)	0.040

Abbreviations: MAF, minor allele frequency; rMDD, recurrent major depressive disorder; SNPs, single-nucleotide polymorphisms.

See main text for trait descriptions and Supplementary Table S8 for results on all diagnoses and traits. Quantitative trait analysis was one-tailed under the hypothesis that an increased burden of minor alleles would reduce scores for cognitive traits and increase scores for anxiety, depression and neuroticism.

References for all quantitative traits are available in the open-access Lothian Birth Cohort protocol paper (Deary *et al.*^60^). Coding: SNPs in protein-coding regions of exons including both synonymous and non-synonymous SNPs. PCon, SNPs within regions of conservation in placental mammals (PhastCon, UCSC). RegPot: SNPs with putative regulatory potential (UCSC, 7 x regulatory potential). CpG: SNPs in CpG islands commonly associated with promoter regions.

a Tested using the Hospital Anxiety Depression Scales.

b The Moray House Test is the general cognitive test—mostly a verbal reasoning, IQ-type test—that was used in the Scottish Mental Survey 1947.

## References

[bib1] Gottesman II, Laursen TM, Bertelsen A, Mortensen PB. Severe mental disorders in offspring with 2 psychiatrically ill parents. Arch Gen Psychiatry 2010; 67: 252–257. 20194825 10.1001/archgenpsychiatry.2010.1

[bib2] Lichtenstein P, Yip BH, Björk C, Pawitan Y, Cannon TD, Sullivan PF et al. Common genetic determinants of schizophrenia and bipolar disorder in Swedish families: a population-based study. Lancet 2009; 373: 234–239. 19150704 10.1016/S0140-6736(09)60072-6 PMC3879718

[bib3] Purcell S, Neale B, Todd-Brown K, Thomas L, Ferreira MA, Bender D et al. PLINK: a tool set for whole-genome association and population-based linkage analyses. Am J Hum Genet 2007; 81: 559–575. 17701901 10.1086/519795 PMC1950838

[bib4] Sullivan PF, Daly MJ, O’Donovan M. Genetic architectures of psychiatric disorders: the emerging picture and its implications. Nat Rev Genet 2012; 13: 537–551. 22777127 10.1038/nrg3240 PMC4110909

[bib5] Blackwood DH, Fordyce A, Walker MT, St Clair DM, Porteous DJ, Muir WJ. Schizophrenia and affective disorders—cosegregation with a translocation at chromosome 1q42 that directly disrupts brain-expressed genes: clinical and P300 findings in a family. Am J Hum Genet 2001; 69: 428–433. 11443544 10.1086/321969 PMC1235314

[bib6] Eykelenboom JE, Briggs GJ, Bradshaw NJ, Soares DC, Ogawa F, Christie S et al. A t(1;11) translocation linked to schizophrenia and affective disorders gives rise to aberrant chimeric DISC1 transcripts that encode structurally altered, deleterious mitochondrial proteins. Hum Mol Genet 2012; 21: 3374–3386. 22547224 10.1093/hmg/dds169 PMC3392113

[bib7] Millar JK, Wilson-Annan JC, Anderson S, Christie S, Taylor MS, Semple CA et al. Disruption of two novel genes by a translocation co-segregating with schizophrenia. Hum Mol Genet 2000; 9: 1415–1423. 10814723 10.1093/hmg/9.9.1415

[bib8] Zhou X, Chen Q, Schaukowitch K, Kelsoe JR, Geyer MA. Insoluble DISC1-Boymaw fusion proteins generated by DISC1 translocation. Mol Psychiatry 2010; 15: 669–672. 20351725 10.1038/mp.2009.127 PMC2891102

[bib9] Johnstone M, Thomson PA, Hall J, McIntosh AM, Lawrie SM, Porteous DJ. DISC1 in schizophrenia: genetic mouse models and human genomic imaging. Schizophrenia Bull 2011; 37: 14–20. 10.1093/schbul/sbq135 PMC3004186 21149852

[bib10] Moens LN, De Rijk P, Reumers J, Van den Bossche MJ, Glassee W, De Zutter S et al. Sequencing of DISC1 pathway genes reveals increased burden of rare missense variants in schizophrenia patients from a northern Swedish population. PLoS ONE 2011; 6: e23450. 21853134 10.1371/journal.pone.0023450 PMC3154939

[bib11] Song W, Li W, Feng J, Heston LL, Scaringe WA, Sommer SS. Identification of high risk DISC1 structural variants with a 2% attributable risk for schizophrenia. Biochem Biophys Res Commun 2008; 367: 700–706. 18164685 10.1016/j.bbrc.2007.12.117

[bib12] Bradshaw NJ, Porteous DJ. DISC1-binding proteins in neural development, signalling and schizophrenia. Neuropharmacology 2012; 62: 1230–1241. 21195721 10.1016/j.neuropharm.2010.12.027 PMC3275753

[bib13] Carless MA, Glahn DC, Johnson MP, Curran JE, Bozaoglu K, Dyer TD et al. Impact of DISC1 variation on neuroanatomical and neurocognitive phenotypes. Mol Psychiatry 2011; 16: 1096–1104, 1063. 21483430 10.1038/mp.2011.37 PMC3135724

[bib14] Song W, Li W, Noltner K, Yan J, Green E, Grozeva D et al. Identification of high risk DISC1 protein structural variants in patients with bipolar spectrum disorder. Neurosci Lett 2010; 486: 136–140. 20850505 10.1016/j.neulet.2010.09.027

[bib15] Williams JM, Beck TF, Pearson DM, Proud MB, Cheung SW, Scott DAA. 1q42 deletion involving DISC1, DISC2, and TSNAX in an autism spectrum disorder. Am J Med Genet A 2009; 149A: 1758–1762. 19606485 10.1002/ajmg.a.32941 PMC2909829

[bib16] Osbun N, Li J, O’Driscoll MC, Strominger Z, Wakahiro M, Rider E et al. Genetic and functional analyses identify DISC1 as a novel callosal agenesis candidate gene. Am J Med Genet A 2011; 155A: 1865–1876. 21739582 10.1002/ajmg.a.34081 PMC5544936

[bib17] Green EK, Grozeva D, Sims R, Raybould R, Forty L, Gordon-Smith K et al. DISC1 exon 11 rare variants found more commonly in schizoaffective spectrum cases than controls. Am J Med Genet B Neuropsychiatr Genet 2011; 156B: 490–492. 21445958 10.1002/ajmg.b.31187

[bib18] Mathieson I, Munafo MR, Flint J. Meta-analysis indicates that common variants at the DISC1 locus are not associated with schizophrenia. Mol Psychiatry 2012; 17: 634–641. 21483435 10.1038/mp.2011.41 PMC3359642

[bib19] Ng MY, Levinson DF, Faraone SV, Suarez BK, DeLisi LE, Arinami T et al. Meta-analysis of 32 genome-wide linkage studies of schizophrenia. Mol Psychiatry 2009; 14: 774–785. 19349958 10.1038/mp.2008.135 PMC2715392

[bib20] Consortium PG. A mega-analysis of genome-wide association studies for major depressive disorder. Mol Psychiatry 2012; 18: 497–511. 22472876 10.1038/mp.2012.21 PMC3837431

[bib21] Ripke S, Sanders AR, Kendler KS, Levinson DF, Sklar P, Holmans PA et al. Genome-wide association study identifies five new schizophrenia loci. Nat Genet 2011; 43: 969–976. 21926974 10.1038/ng.940 PMC3303194

[bib22] Sklar P, Ripke S, Scott LJ, Andreassen OA, Cichon S, Craddock N et al. Large-scale genome-wide association analysis of bipolar disorder identifies a new susceptibility locus near ODZ4. Nat Genet 2011; 43: 977–983. 21926972 10.1038/ng.943 PMC3637176

[bib23] Crowley JJ, Hilliard CE, Kim Y, Morgan MB, Lewis LR, Muzny DM et al. Deep resequencing and association analysis of schizophrenia candidate genes. Mol Psychiatry 2012; 18: 138–140. 22472875 10.1038/mp.2012.28 PMC3577417

[bib24] Jia P, Wang L, Fanous AH, Pato CN, Edwards TL, Zhao Z. Network-assisted investigation of combined causal signals from genome-wide association studies in schizophrenia. PLoS Comput Biol 2012; 8: e1002587. 22792057 10.1371/journal.pcbi.1002587 PMC3390381

[bib25] Ayalew M, Le-Niculescu H, Levey DF, Jain N, Changala B, Patel SD et al. Convergent functional genomics of schizophrenia: from comprehensive understanding to genetic risk prediction. Mol Psychiatry 2012; 17: 887–905. 22584867 10.1038/mp.2012.37 PMC3427857

[bib26] Le-Niculescu H, Patel SD, Bhat M, Kuczenski R, Faraone SV, Tsuang MT et al. Convergent functional genomics of genome-wide association data for bipolar disorder: comprehensive identification of candidate genes, pathways and mechanisms. Am J Med Genet B Neuropsychiatr Genet 2009; 150B: 155–181. 19025758 10.1002/ajmg.b.30887

[bib27] Tiwary BK. The severity of mental disorders is linked to interaction among candidate genes. Integr Biol (Camb) 2012; 4: 1096–1101. 22777684 10.1039/c2ib20066j

[bib28] Insel TR. Rethinking schizophrenia. Nature 2010; 468: 187–193. 21068826 10.1038/nature09552

[bib29] Porteous DJ, Millar JK, Brandon NJ, Sawa A. DISC1 at 10: connecting psychiatric genetics and neuroscience. Trends Mol Med 2011; 17: 699–706. 22015021 10.1016/j.molmed.2011.09.002 PMC3253483

[bib30] Brandon NJ, Sawa A. Linking neurodevelopmental and synaptic theories of mental illness through DISC1. Nat Rev Neurosci 2011; 12: 707–722. 22095064 10.1038/nrn3120 PMC3954824

[bib31] Soares DC, Carlyle BC, Bradshaw NJ, Porteous DJ. DISC1: Structure, Function, and Therapeutic Potential for Major Mental Illness. ACS Chem Neurosci 2011; 2: 609–632. 22116789 10.1021/cn200062k PMC3222219

[bib32] Mao Y, Ge X, Frank CL, Madison JM, Koehler AN, Doud MK et al. Disrupted in schizophrenia 1 regulates neuronal progenitor proliferation via modulation of GSK3beta/beta-catenin signaling. Cell 2009; 136: 1017–1031. 19303846 10.1016/j.cell.2008.12.044 PMC2704382

[bib33] Millar JK, Pickard BS, Mackie S, James R, Christie S, Buchanan SR et al. DISC1 and PDE4B are interacting genetic factors in schizophrenia that regulate cAMP signaling. Science 2005; 310: 1187–1191. 16293762 10.1126/science.1112915

[bib34] Hashimoto R, Numakawa T, Ohnishi T, Kumamaru E, Yagasaki Y, Ishimoto T et al. Impact of the DISC1 Ser704Cys polymorphism on risk for major depression, brain morphology and ERK signaling. Hum Mol Genet 2006; 15: 3024–3033. 16959794 10.1093/hmg/ddl244

[bib35] Kamiya A, Tomoda T, Chang J, Takaki M, Zhan C, Morita M et al. DISC1-NDEL1/NUDEL protein interaction, an essential component for neurite outgrowth, is modulated by genetic variations of DISC1. Hum Mol Genet 2006; 15: 3313–3323. 17035248 10.1093/hmg/ddl407

[bib36] Burdick KE, Kamiya A, Hodgkinson CA, Lencz T, DeRosse P, Ishizuka K et al. Elucidating the relationship between *DISC1*, *NDEL1*, and *NDE1* and the risk for schizophrenia: Evidence of epistasis and competitive binding. Hum Mol Genet 2008; 17: 2462–2473. 18469341 10.1093/hmg/ddn146 PMC2486442

[bib37] Leliveld SR, Hendriks P, Michel M, Sajnani G, Bader V, Trossbach S et al. Oligomer assembly of the C-terminal DISC1 domain (640–854) is controlled by self-association motifs and disease-associated polymorphism S704C. Biochemistry 2009; 48: 7746–7755. 19583211 10.1021/bi900901e

[bib38] Eastwood SL, Hodgkinson CA, Harrison PJ. DISC-1 Leu607Phe alleles differentially affect centrosomal PCM1 localization and neurotransmitter release. Mol Psychiatry 2009; 14: 556–557. 19455170 10.1038/mp.2009.13

[bib39] Atkin TA, MacAskill AF, Brandon NJ, Kittler JT. Disrupted in Schizophrenia-1 regulates intracellular trafficking of mitochondria in neurons. Mol Psychiatry 2011; 16: 122–124, 121. 21079610 10.1038/mp.2010.110

[bib40] Eastwood SL, Walker M, Hyde TM, Kleinman JE, Harrison PJ. The DISC1 Ser704Cys substitution affects centrosomal localization of its binding partner PCM1 in glia in human brain. Hum Mol Genet 2010; 19: 2487–2496. 20360304 10.1093/hmg/ddq130 PMC2876891

[bib41] Singh KK, De Rienzo G, Drane L, Mao Y, Flood Z, Madison J et al. Common DISC1 polymorphisms disrupt Wnt/GSK3beta signaling and brain development. Neuron 2011; 72: 545–558. 22099458 10.1016/j.neuron.2011.09.030 PMC3387684

[bib42] Millar JK, Pickard BS, Mackie S, James R, Christie S, Buchanan SR et al. DISC1 and PDE4B are interacting genetic factors in schizophrenia that regulate cAMP signaling.[see comment]. Science 2005; 310: 1187–1191. 16293762 10.1126/science.1112915

[bib43] Mao Y, Ge X, Frank CL, Madison JM, Koehler AN, Doud MK et al. Disrupted in Schizophrenia 1 regulates neuronal progenitor proliferation via modulation of GSK3^2^/^2^-catenin signaling. Cell 2009; 136: 1017–1031. 19303846 10.1016/j.cell.2008.12.044 PMC2704382

[bib44] Malavasi EL, Ogawa F, Porteous DJ, Millar JK. DISC1 variants 37W and 607F disrupt its nuclear targeting and regulatory role in ATF4-mediated transcription. Hum Mol Genet 2012; 21: 2779–2792. 22422769 10.1093/hmg/dds106 PMC3363331

[bib45] Millar JK, Christie S, Semple CA, Porteous DJ. Chromosomal location and genomic structure of the human translin-associated factor X gene (TRAX; TSNAX) revealed by intergenic splicing to DISC1, a gene disrupted by a translocation segregating with schizophrenia. Genomics 2000; 67: 69–77. 10945471 10.1006/geno.2000.6239

[bib46] Hennah W, Porteous D. The DISC1 pathway modulates expression of neurodevelopmental, synaptogenic and sensory perception genes. PLoS ONE 2009; 4: e4906. 19300510 10.1371/journal.pone.0004906 PMC2654149

[bib47] Thomson PA, Macintyre DJ, Hamilton G, Dominiczak A, Smith BH, Morris A et al. Association of DISC1 variants with age of onset in a population-based sample of recurrent major depression. Mol Psychiatry 2012; 18: 745–747. 22869032 10.1038/mp.2012.117

[bib48] Walker RM, Hill AE, Newman AC, Hamilton G, Torrance HS, Anderson SM et al. The DISC1 promoter: characterization and regulation by FOXP2. Hum Mol Genet 2012; 21: 2862–2872. 22434823 10.1093/hmg/dds111

[bib49] Li H, Ruan J, Durbin R. Mapping short DNA sequencing reads and calling variants using mapping quality scores. Genome Res 2008; 18: 1851–1858. 18714091 10.1101/gr.078212.108 PMC2577856

[bib50] Chelala C, Khan A, Lemoine NR. SNPnexus: a web database for functional annotation of newly discovered and public domain single nucleotide polymorphisms. Bioinformatics 2009; 25: 655–661. 19098027 10.1093/bioinformatics/btn653 PMC2647830

[bib51] Tempel S. Using and understanding RepeatMasker. Methods Mol Biol 2012; 859: 29–51. 22367864 10.1007/978-1-61779-603-6_2

[bib52] Ferrer-Costa C, Gelpi JL, Zamakola L, Parraga I, de la Cruz X, Orozco M. PMUT: a web-based tool for the annotation of pathological mutations on proteins. Bioinformatics 2005; 21: 3176–3178. 15879453 10.1093/bioinformatics/bti486

[bib53] Thomas PD, Campbell MJ, Kejariwal A, Mi H, Karlak B, Daverman R et al. PANTHER: a library of protein families and subfamilies indexed by function. Genome Res 2003; 13: 2129–2141. 12952881 10.1101/gr.772403 PMC403709

[bib54] Adzhubei IA, Schmidt S, Peshkin L, Ramensky VE, Gerasimova A, Bork P et al. A method and server for predicting damaging missense mutations. Nat Methods 7: 248–249. 20354512 10.1038/nmeth0410-248 PMC2855889

[bib55] Blom N, Gammeltoft S, Brunak S. Sequence and structure-based prediction of eukaryotic protein phosphorylation sites. J Mol Biol 1999; 294: 1351–1362. 10600390 10.1006/jmbi.1999.3310

[bib56] Purcell S, Neale B, Todd-Brown K, Thomas L, Ferreira MA, Bender D et al. PLINK: a tool set for whole-genome association and population-based linkage analyses. Am J Hum Genet 2007; 81: 559–575. 17701901 10.1086/519795 PMC1950838

[bib57] Petersen CGJ. The yearly immigration of young plaice into the Limfjord from the German Sea. Danish Biol Station 1896; 6: 1–77.

[bib58] Lincoln FC. Calculating Waterfowl Abundances on the Basis of Banding Returns 1930, US Department of Agriculture Circular 118, 1–4.

[bib59] Chapman DG. Some properties of the hypergeometric distribution with applications to zoological sample censuses. University of California Press: Berkeley, 1951.

[bib60] Deary IJ, Gow AJ, Taylor MD, Corley J, Brett C, Wilson V et al. The Lothian Birth Cohort 1936: a study to examine influences on cognitive ageing from age 11 to age 70 and beyond. BMC Geriatr 2007; 7: 28. 18053258 10.1186/1471-2318-7-28 PMC2222601

[bib61] Consortium TGP. A map of human genome variation from population-scale sequencing. Nature 2010; 467: 1061–1073. 20981092 10.1038/nature09534 PMC3042601

[bib62] Purcell SM, Wray NR, Stone JL, Visscher PM, O’Donovan MC, Sullivan PF et al. Common polygenic variation contributes to risk of schizophrenia and bipolar disorder. Nature 2009; 460: 748–752. 19571811 10.1038/nature08185 PMC3912837

[bib63] Wray NR, Pergadia ML, Blackwood DH, Penninx BW, Gordon SD, Nyholt DR et al. Genome-wide association study of major depressive disorder: new results, meta-analysis, and lessons learned. Mol Psychiatry 2012; 17: 36–48. 21042317 10.1038/mp.2010.109 PMC3252611

[bib64] Luciano M, Gow AJ, Harris SE, Hayward C, Allerhand M, Starr JM et al. Cognitive ability at age 11 and 70 years, information processing speed, and APOE variation: the Lothian Birth Cohort 1936 study. Psychol Aging 2009; 24: 129–138. 19290744 10.1037/a0014780

[bib65] Davies G, Tenesa A, Payton A, Yang J, Harris SE, Liewald D et al. Genome-wide association studies establish that human intelligence is highly heritable and polygenic. Mol Psychiatry 2011; 16: 996–1005. 21826061 10.1038/mp.2011.85 PMC3182557

[bib66] Deary IJ, Yang J, Davies G, Harris SE, Tenesa A, Liewald D et al. Genetic contributions to stability and change in intelligence from childhood to old age. Nature 2012; 482: 212–215. 22258510 10.1038/nature10781

[bib67] The 1000 Genomes Project Consortium. A map of human genome variation from population-scale sequencing. Nature 2010; 467: 1061–1073. 20981092 10.1038/nature09534 PMC3042601

[bib68] Song W, Li W, Feng J, Heston LL, Scaringe WA, Sommer SS. Identification of high risk DISC1 structural variants with a 2% attributable risk for schizophrenia. Biochem Biophys Res Commun 2008; 367: 700–706. 18164685 10.1016/j.bbrc.2007.12.117

[bib69] Song W, Li W, Noltner K, Yan J, Green E, Grozeva D et al. Identification of high risk DISC1 protein structural variants in patients with bipolar spectrum disorder. Neurosci Lett 2010; 486: 136–140. 20850505 10.1016/j.neulet.2010.09.027

[bib70] Hennah W, Thomson P, McQuillin A, Bass N, Loukola A, Anjorin A et al. DISC1 association, heterogeneity and interplay in schizophrenia and bipolar disorder. Mol Psychiatry 2009; 14: 865–873. 18317464 10.1038/mp.2008.22

[bib71] Sawamura N, Ando T, Maruyama Y, Fujimuro M, Mochizuki H, Honjo K et al. Nuclear DISC1 regulates CRE-mediated gene transcription and sleep homeostasis in the fruit fly. Mol Psychiatry 2008; 13: 1138–1148, 1069. 18762802 10.1038/mp.2008.101 PMC2727926

[bib72] Murdoch H, Mackie S, Collins DM, Hill EV, Bolger GB, Klussmann E et al. Isoform-selective susceptibility of DISC1/phosphodiesterase-4 complexes to dissociation by elevated intracellular cAMP levels. J Neurosci 2007; 27: 9513–9524. 17728464 10.1523/JNEUROSCI.1493-07.2007 PMC6673124

[bib73] Singh KK, De Rienzo G, Drane L, Mao Y, Flood Z, Madison J et al. Common DISC1 polymorphisms disrupt Wnt/GSK3beta signaling and brain development. Neuron 72: 545–558. 22099458 10.1016/j.neuron.2011.09.030 PMC3387684

[bib74] Kiezun A, Garimella K, Do R, Stitziel NO, Neale BM, McLaren PJ et al. Exome sequencing and the genetic basis of complex traits. Nat Genet 2012; 44: 623–630. 22641211 10.1038/ng.2303 PMC3727622

[bib75] Sullivan PF, Wells JE, Joyce PR, Bushnell JA, Mulder RT, Oakley-Browne MA. Family history of depression in clinic and community samples. J Affect Disord 1996; 40: 159–168. 8897115 10.1016/0165-0327(96)00056-0

[bib76] Eaton WW, Anthony JC, Gallo J, Cai G, Tien A, Romanoski A et al. Natural history of Diagnostic Interview Schedule/DSM-IV major depression. The Baltimore Epidemiologic Catchment Area follow-up. Arch Gen Psychiatry 1997; 54: 993–999. 9366655 10.1001/archpsyc.1997.01830230023003

[bib77] Solomon DA, Keller MB, Leon AC, Mueller TI, Shea MT, Warshaw M et al. Recovery from major depression. A 10-year prospective follow-up across multiple episodes. Arch Gen Psychiatry 1997; 54: 1001–1006. 9366656 10.1001/archpsyc.1997.01830230033005

[bib78] Colantuoni C, Lipska BK, Ye T, Hyde TM, Tao R, Leek JT et al. Temporal dynamics and genetic control of transcription in the human prefrontal cortex. Nature 2011; 478: 519–523. 22031444 10.1038/nature10524 PMC3510670

[bib79] Bernstein BE, Birney E, Dunham I, Green ED, Gunter C, Snyder M. An integrated encyclopedia of DNA elements in the human genome. Nature 2012; 489: 57–74. 22955616 10.1038/nature11247 PMC3439153

[bib80] Niculescu AB, Le-Niculescu H. The *P*-value illusion: how to improve (psychiatric) genetic studies. Am J Med Genet B Neuropsychiatr Genet 2010; 153B: 847–849. 20301110 10.1002/ajmg.b.31076

