# Supplementary material for: 708 Common and 2010 rare DISC1 locus variants identified in 1542 subjects: analysis for association with psychiatric disorder and cognitive traits
Source: Mol Psychiatry. 2013 Jun 4;19(6):668–75. doi: 10.1038/mp.2013.68 (PMC4031635; doi:10.1038/mp.2013.68)
Supplement: Supplementary Material [file mp201368x1.pdf]

Supplemental Material: 708 common and 2,010 rare *DISC1* locus variants identified in 1,542 subjects: analysis for association with psychiatric disorder and cognitive traits.

Human samples

All clinically-ascertained participants gave signed informed consent to the study which had the approval of the appropriate Research Ethics Committee. All participants were from the Scottish population. The schizophrenia, bipolar and rMDD cases for sequence analysis were hospital inpatients and outpatients, as previously described.[1] The Lothian Birth Cohort of 1936 (LBC1936) has been described previously.[2,3] In sum, 1,542 individuals were sequenced, comprising 240 cases of schizophrenia, 221 cases of bipolar disorder, 192 cases of recurrent major depressive disorder (rMDD) and 889 members of LBC1936. The replication study drew upon 466 additional cases of rMDD referred from primary care to a hospital out-patient clinic, plus 600 cases of rMDD and 645 cases of MDD drawn from the Generation Scotland: Scottish Family Health Study (GS:SFHS), a population and family based cohort recruited through primary care receiving intensive phenotyping for health related quantitative traits.[4] Ethical approval for the GS:SFHS was obtained from the Tayside Committee on Medical Research Ethics (on behalf of the National Health Service). GS:SFHS participants were assessed for MDD and rMDD using the SCID, Structured Clinical Interview for DSM-IV Axis I Disorders Schedule. The 4,017 control samples were GS:SFHS participants who did not trigger a SCID and had no diagnosis of mental disorder after screening. GS:SFHS study participants gave signed consent for participation in their respective cohorts and for genetic analyses of their DNA.

Genomic template DNA for sequencing

Genomic DNA purified from blood samples was whole genome amplified to provide template DNA for long-range PCR. To mitigate uneven genome representation, three separate aliquots of 100 ng genomic DNA were whole genome amplified from each individual using the REPLI-g Midi Kit (QIAGEN), and subsequently pooled prior to use in long-range PCR.

Long-range PCR amplification

Long-range PCR amplification of 528 Kb of *TRAX/DISC1* (defined as hg18 chr1:229723339-230251606; hg19 chr1:231656716-232184983) from genomic DNA was performed with

each sample using a set of overlapping primer pairs, designed using Primer3 software.[5,6] Long range PCR amplification used rTth DNA polymerase, XL (Applied Biosystems), producing tiling amplicons ranging from 3.8 to 10.9 Kb, and overlapping on average by 1 Kb. Up to 96 PCRs were performed per sample to selectively amplify the TRAX/DISC1 locus. Each reaction contained 100 ng of template DNA, 0.16 µM forward primer, 0.16 µM reverse primer, 800 µM dNTP mix, 1.1 mM magnesium acetate, and 1 U of rTth DNA polymerase, XL. The reactions were performed in Tetrad 2 thermal cyclers (Bio-Rad) using two-step PCR for 37 cycles according to the manufacturer's guidelines for rTth DNA polymerase, XL. Following PCR, 3 µL from each reaction were analysed using agarose gel electrophoresis (1% agarose in 1X TAE, 0.5 µg/mL ethidium bromide). Samples from one entire 96 well plate of PCR were analysed simultaneously using a single gel to determine relative amounts of amplicons. The results from agarose gel electrophoresis were used to normalize the amplicons from a given sample during amplicon pooling. Pooled amplicons were purified using AMPure (Beckman Coulter Genomics) to remove excess dNTPs, primers, and salts. The purified amplicon pools were then quantified using the NanoDrop 1000 (Thermo Scientific) or the Quant-iT High Sensitivity DNA Assay Kit and diluted to 1 µg DNA in 80 µL of 10 mM Tris-HCl (pH 8.5) prior to being processed into Illumina paired-end libraries.

Library barcoding and sequencing

Each *TRAX/DISC1* amplicon pool was mechanically fragmented by nebulisation or adaptive focused acoustics (Covaris S2 System) and end-repaired using T4 DNA polymerase, Klenow fragment, and T4 polynucleotide kinase (New England Biolabs), then treated with Klenow fragment (3' – 5' exonuclease) (New England Biolabs) to add a single 3' deoxyA overhang and ligated to standard Illumina paired-end adapters or to custom barcode paired-end adapters. Each custom barcode adapter included a unique 5-base sequence that allowed library identification following multiplexed sequencing (SOM Suppl Table 2). To maximize sample throughput, uniquely barcoded samples were pooled and size-selected in 50 bp windows between 200 - 400 bp on certified low range ultra agarose gels (Bio-Rad; 2% in 1X TAE with 0.5 µg/mL ethidium bromide). Each pooled library was PCR enriched using Phusion DNA polymerase in HF buffer (New England Biolabs) for 14 cycles and quantified using the NanoDrop 1000 (Thermo Scientific).

Production sequencing

Production sequencing was performed using Illumina GAII or GAIIx instruments with paired-end modules and using 36-base read lengths, multiplexed at two to six samples per lane. Additional sequence data post-production phase were obtained using Illumina GAIIx or HiSeq 2000 instruments. All individuals were sequenced to >80% coverage at ≥30-fold read depth.

All individuals were sequenced to >80% coverage at ≥30-fold read depth. There was no evidence for sequencing bias between cases and controls (Supplementary Figure S1). In sum, we report 2,718 validated sequence SNPs, of which >60% are novel (1000 Genomes release 20100804 merged 2 of 4 intersection and dbSNP 132) (Table 1 and Supplementary Figure S2). We conducted a visual check on all SNPs called by MAQ and used ABI 3730 Sanger chemistry sequencing to validate the 2,947 variants appearing at a frequency of <1%, observing a validation rate of 68.2%, thus confirming 2,010 rare variants. A proportion of the non-validated calls (~35%) could be ascribed to PCR amplification artefacts (data not shown). The false positive rate found in this study is not unexpected, even a low rate of sequencing error can quickly lead to the detection of singletons that outnumber actual rare variants. For example, using a 1 in 300,000 error rate over 450 Kb for 1,500 samples, we would expect 2,250 false positives. Given an expectation of ~1,550 true variants for the same region, that is a false positive rate of 59%.

Of the 2,718 observed single nucleotide variants, 826 were reported in the European subset of the 1000 Genomes Project[7] (20100804 2 of 4 intersection), with 1,031 reported in the combined set or in dbSNP 132.

Initial sequence analysis

The Illumina pipeline (v1.3/v1.4.0/v1.5/v1.6) was used for primary analysis of the sequence data, and to produce fastq sequence files. An in-house Perl script was used to sort multiplexed sequence reads into separate files according to their 5’ barcode sequences. After removal of the barcodes the read length was 31 bases.

Sequence alignment and SNP calling

The demultiplexed pass-filter data were aligned to the hg18 reference, and repeat sequence elements were marked with RepeatMasker[8] (http://repeatmasker.org). Read pairs with identical outer coordinates were removed to avoid spurious SNP calls from duplicate library

fragments. We then recalibrated the data against a validated subset of variants to determine the appropriate MAQ quality score cut-offs.[9]

MAQ software[9] was used for SNP calling. Both the sequence reads and the reference sequence (consisting of the *TRAX/DISC1* target region downloaded from UCSC hg18) were converted to binary format. Paired reads were then aligned to the target region using MAQ match, allowing at least 2 mismatches. MAQ uses base quality values to choose the best alignment and also assigns a mapping quality score for each alignment. Read duplicates were filtered using MAQ rmdup, which removes read pairs that have identical start and end points. Such duplicates are likely to be PCR copies of the same library fragment, and retaining them for variant calling decreases the accuracy of consensus genotype calls. The alignment files were subsequently parsed by MAQ pileup, to determine base coverage at each position in the target. A Perl script was used to determine the percentage of the target region covered to 30-fold depth or greater for each sample. Initial oversampling was planned to increase coverage in regions that showed lower representation, as well as to increase confidence for SNP calling. MAQ assemble was used to build the consensus sequences and qualities from the mapped reads. The MAQ Perl script cns2snp was used to extract the SNP sites, the SNP calls, the read depths and the consensus quality scores. These sites were filtered with maq.pl SNPfilter to exclude any site with a read depth below 20-fold.

The Accession Numbers for the finished sequence are NCBI ss472328925 - ss472331023.

Secondary genotype quality control

All samples had prior genotype information for a number SNPs throughout the DISC1 region, either from whole-genome SNP typing or previous studies on DISC1 (range 12 to 171 per sample, average of 102). This was used to audit and confirm the pipeline and for quality control. For any sample with one or more genotype mismatches between the SNP typing and sequence data, the additive genetic relationship between the SNP genotypes and all sequenced individuals was estimated,[10] confirming that the sequence data for the putative sample matched the SNP data more highly than any other sample. The additive genetic relationship between all samples was similarly estimated and one of any pair of samples showing a cryptic relatedness was removed from the study (with preference for keeping the sample with greater sequence coverage). When multiple sequencing runs were available for an individual, SNP genotypes were called on each sample separately and any mismatched SNP call set as missing.

Bioinformatics annotation of SNPs

The coordinates of non-reference sequence bases were queried in the MySQL tables of the UCSC Human Genome Browser NCBI36/hg18 (March, 2006 http://genome.ucsc.edu). SNPs located in regions identified as repeats (Variation & repeats; Repeat masker) were removed from further analysis due to inherent difficulties in mapping 31-base reads in repetitive regions. Known SNPs including those identified in the 1000 Genomes Project[7] were identified.

The potential functional effects of all remaining variants were assessed by querying further UCSC tracks (see Supplementary Table 7) and using the SNPnexus SNP annotation tool (http://www.snp-nexus.org).[11] SNPnexus interrogates multiple primary data sources from Ensembl and UCSC. SNPnexus uses MySQL tables from the March 2006 GenBank freeze assembled by NCBI (http://hgdownload.cse.ucsc.edu/goldenPath/hg18/database/, hg18, Build 36,) and EnsemblMart_48 (ftp://ftp.ensembl.org/pub/release-48/mysql/ensembl_mart__48). It links to the Genetic Association Database GAD data file and miRBase data from the database FTP site (http://microrna.sanger.ac.uk/sequences/ftp.shtml). Structural variability data is derived from the Database of Genomic Variants (DGV) (http://projects.tcag.ca/variation/) via UCSC. SNPnexus is updated on a regular monthly basis. Non-coding variants were annotated using the UCSC table browser for the following tracks: 'RepeatMasker', 'CpG island', 'TFBS conserved', '7x Reg Potential' and/or '28-Way Most conserved – PlacMammal' [http://genome.ucsc.edu/].

Assessment of coding variants

Sequence variants classified as coding were mapped to the DISC1 L isoform and potential pathogenicity ascribed using Pmut,[12] Panther[13] and PolyPhen-2.[14] The coding sequence variants were mapped onto a list of known curated DISC1-interactor binding sites and with other functional elements (e.g. nuclear localisation and phosphorylation, Supplementary Table S2).[15] {Blom, 1999 #208}

Single SNP association:

SNPs with greater than 20% missing data and Hardy-Weinberg equilibrium *P*-values less than 0.00001 were excluded from the analysis. Association tests were performed on all

SNPs regardless of frequency as the inclusion of all rare variants has little effect on the multiple testing burden for the region. Case-control association was tested using Fisher's exact test on the combined case samples as well as individually for each of schizophrenia, bipolar and recurrent major depression. Quantitative trait association analyses were performed by linear regression of the trait residuals on the number of minor alleles at each SNP, with empirical significance estimated by permutation to avoid issues with the test statistic distribution caused by the combination of rare variants and slight deviations from normality in the data. All analysis was performed using PLINK.[16] Region-wide significance thresholds were estimated by evaluating the distribution of the most-significant SNP *P*-value in the region from 10,000 permutations of the data.

Burden of SNP variants:

Two tests were performed to assess the burden of variants over the entire region on the traits being examined. The first test (labelled BURDEN) calculated the burden of variants in each individual as the sum of the number of minor alleles observed across all sites regardless of their minor allele frequency.[16] For sites with missing genotypes, a contribution equal to the expected number of variants (twice the minor allele frequency) was added to the burden value. In the case-control analysis, the difference in average burden for cases and controls was tested. For quantitative traits, the correlation between the burden and the trait of interest was tested with the *a priori* assumption of directionality of effect on the trait (an increase in rare variant burden was assumed to decrease measures of IQ and reading ability, but increase depression, anxiety and neuroticism measures). A second test of burden (VTTEST)[17] was performed where the maximum minor allele frequency for inclusion in the burden measure is determined by finding the frequency at which the difference (standardised) in average burden between cases and controls is maximised (or the correlation for quantitative trait data). The empirical p-value of all test statistics was estimated with 1000 permutations of the phenotypes data across the genotypes.

The burden analysis was performed on all SNPs and for subsets based on biological annotation of variants being exonic, in coding regions, non-synonymous, in conserved regions, in regions of regulatory potential, in transcription factor binding sites and in CpG islands (see Bioinformatics annotation of SNPs above).

Supplementary references

1. Blackwood DH, Fordyce A, Walker MT, St Clair DM, Porteous DJ, Muir WJ. Schizophrenia and affective disorders--cosegregation with a translocation at chromosome 1q42 that directly disrupts brain-expressed genes: clinical and P300 findings in a family. *American Journal of Human Genetics* 2001; **69:** 428-433.

2. Deary IJ, Gow AJ, Taylor MD, Corley J, Brett C, Wilson V *et al.* The Lothian Birth Cohort 1936: a study to examine influences on cognitive ageing from age 11 to age 70 and beyond. *BMC Geriatr* 2007; **7:** 28.

3. Deary IJ, Yang J, Davies G, Harris SE, Tenesa A, Liewald D *et al.* Genetic contributions to stability and change in intelligence from childhood to old age. *Nature* 2012; **482:** 212-215.

4. Smith BH, Campbell H, Blackwood D, Connell J, Connor M, Deary IJ *et al.* Generation Scotland: the Scottish Family Health Study; a new resource for researching genes and heritability. *BMC Med Genet* 2006; **7:** 74.

5. Koressaar T, Remm M. Enhancements and modifications of primer design program Primer3. *Bioinformatics* 2007; **23:** 1289-1291.

6. Rozen S, Skaletsky H, Marszalek JD, Minx PJ, Cordum HS, Waterston RH *et al.* Abundant gene conversion between arms of palindromes in human and ape Y chromosomes. *Nature* 2003; **423:** 873-876.

7. Consortium TGP. A map of human genome variation from population-scale sequencing. *Nature* 2010; **467:** 1061-1073.

8. Tempel S. Using and understanding RepeatMasker. *Methods Mol Biol* 2012; **859:** 29-51.

9. Li H, Ruan J, Durbin R. Mapping short DNA sequencing reads and calling variants using mapping quality scores. *Genome Res* 2008; **18:** 1851-1858.

10. Yang L, Zhu H, Guo W, Zhang T. Molecular cloning and characterization of five genes encoding pentatricopeptide repeat proteins from Upland cotton (Gossypium hirsutum L.). *Mol Biol Rep* 2010; **37:** 801-808.

11. Chelala C, Khan A, Lemoine NR. SNPnexus: a web database for functional annotation of newly discovered and public domain single nucleotide polymorphisms. *Bioinformatics* 2009; **25:** 655-661.

12. Ferrer-Costa C, Gelpi JL, Zamakola L, Parraga I, de la Cruz X, Orozco M. PMUT: a web-based tool for the annotation of pathological mutations on proteins. *Bioinformatics* 2005; **21:** 3176-3178.

13. Thomas PD, Campbell MJ, Kejariwal A, Mi H, Karlak B, Daverman R *et al.* PANTHER: a library of protein families and subfamilies indexed by function. *Genome Res* 2003; **13:** 2129-2141.

14. Adzhubei IA, Schmidt S, Peshkin L, Ramensky VE, Gerasimova A, Bork P *et al.* A method and server for predicting damaging missense mutations. *Nat Methods* 2010; **7:** 248-249.

15. Soares DC, Carlyle BC, Bradshaw NJ, Porteous DJ. DISC1: Structure, Function, and Therapeutic Potential for Major Mental Illness. *ACS Chem Neurosci* 2011; **2:** 609-632.

16. Purcell S, Neale B, Todd-Brown K, Thomas L, Ferreira MA, Bender D *et al.* PLINK: a tool set for whole-genome association and population-based linkage analyses. *American Journal of Human Genetics* 2007; **81:** 559-575.

17. Price AL, Kryukov GV, de Bakker PI, Purcell SM, Staples J, Wei LJ *et al.* Pooled association tests for rare variants in exon-resequencing studies. *Am J Hum Genet* 2010; **86:** 832-838.

Supplementary Figures

Supplementary Figure S1. Locus-wide analysis of sequence completeness in cases and controls.

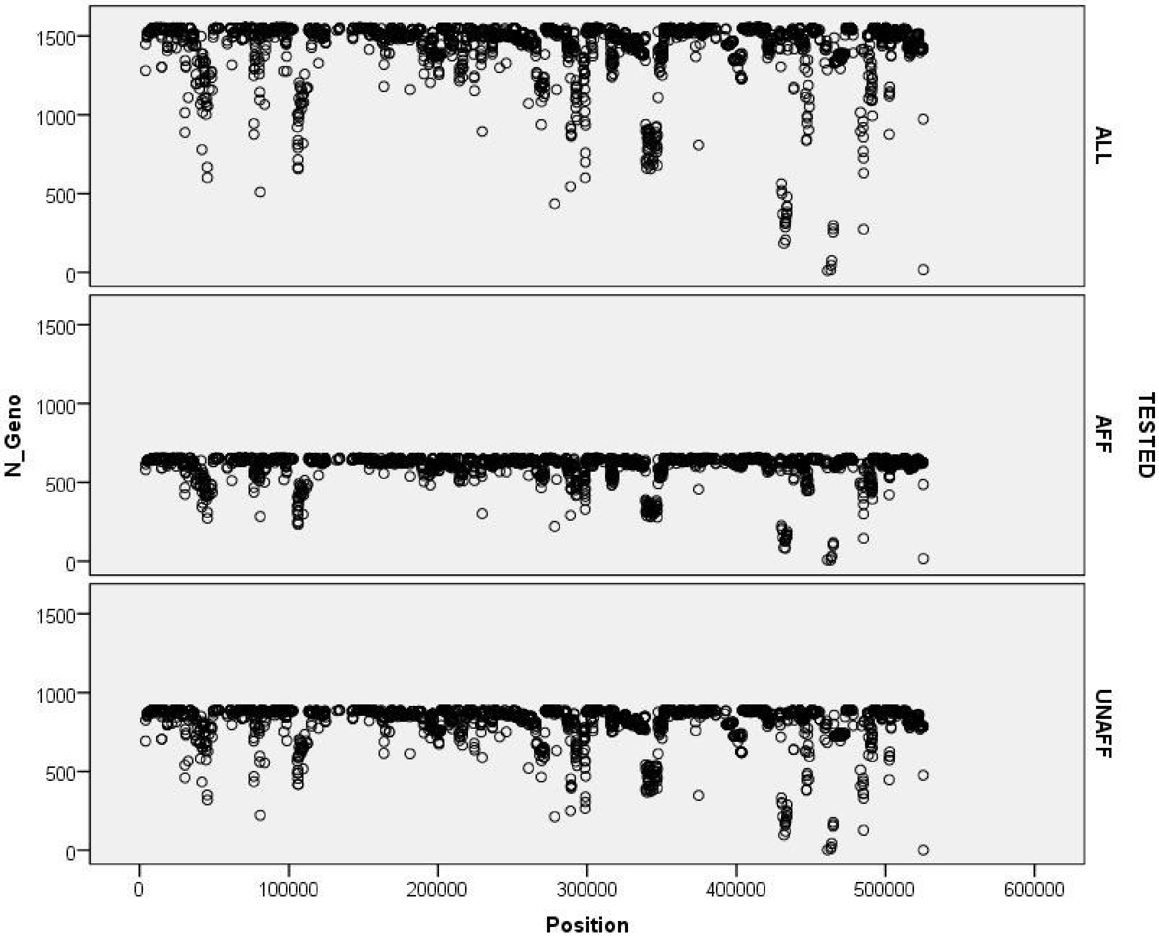

The number of individuals genotyped at each SNP in the region are shown for both cases (AFF) and controls (UNAFF) separately and combined (ALL). The correlation between the number of individuals genotyped in cases and controls across all SNPs in the region is 0.920.

Supplementary Figure S2. Allele frequencies in the 1000 Genomes Project and LBC1936 controls.

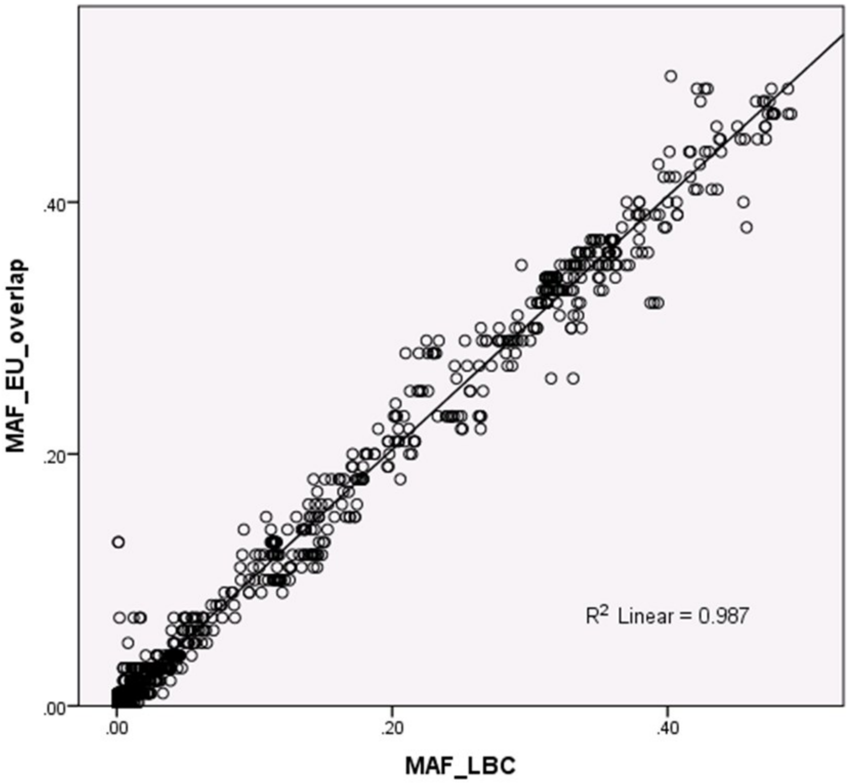

Comparison of the allele frequencies (AF) of SNPs identified in the European subset of the 1000 Genomes Project and LBC1936 healthy adults in this study are very highly correlated, r2=0.965. The exceptional SNP, marked with an arrow, is within a poly(T) mononucleotide repeat. The allele frequencies of cases versus controls within our study sample are similarly very highly correlated, r2=0.991.

Supplementary Figure S3. Annotation of exonic SNPs on the predicted protein domains of (A) *TRAX* and (B) *DISC1*.

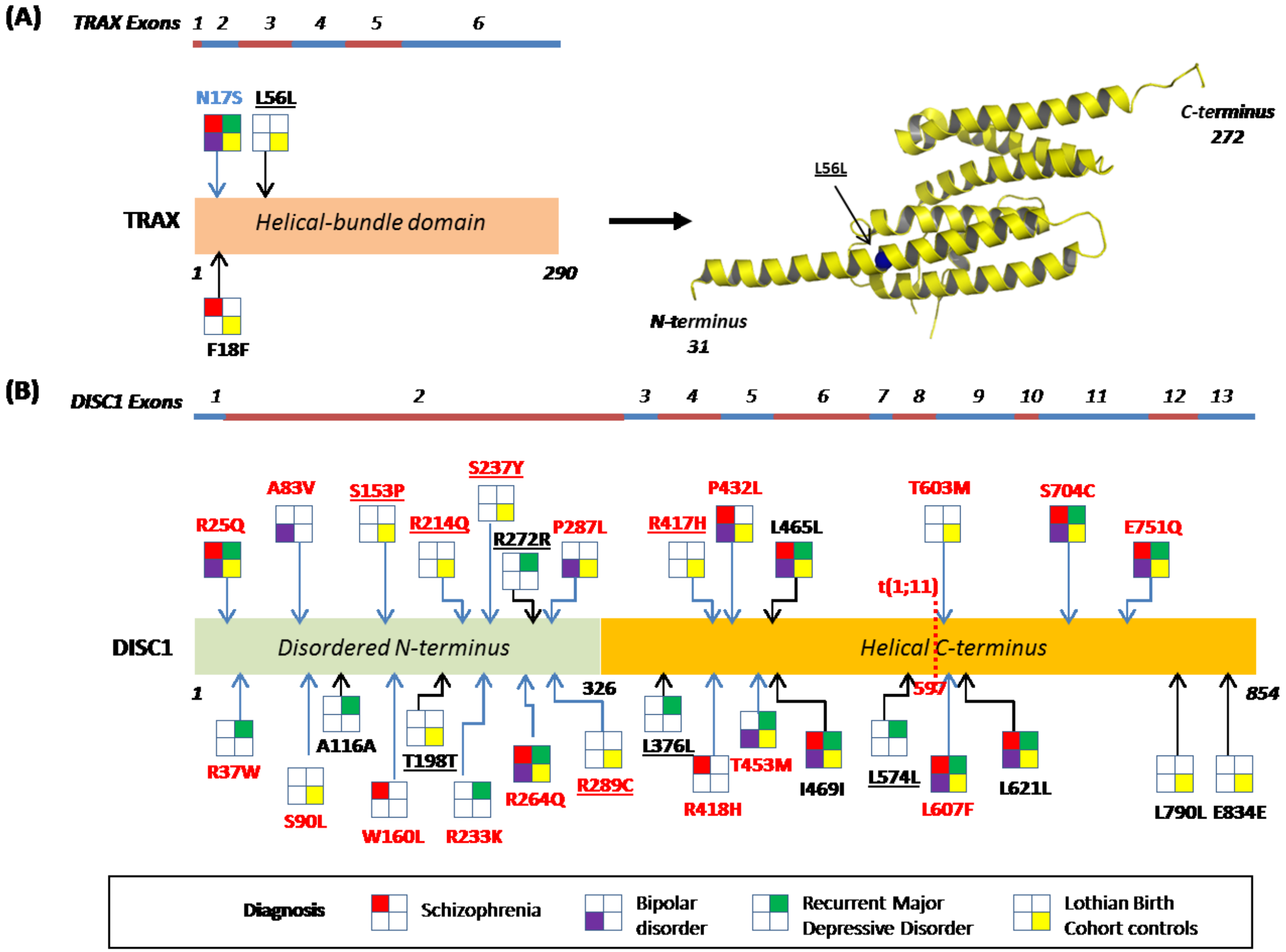

SOM Supplementary Figure 3. Annotation of exonic SNPs on the predicted protein domains of TRAX (A) and DISC1 (B). The position of exonic SNPs and diagnostic class in which they were identified are shown: synonymous (black) and non-synonymous (red). SNPs not seen in the 1000 Genomes Project, the Exome Variant server or previously published sequencing projects are underlined.

Supplementary Figure S4. Segregation of rs16856199 in families affected with recurrent major depressive disorder.

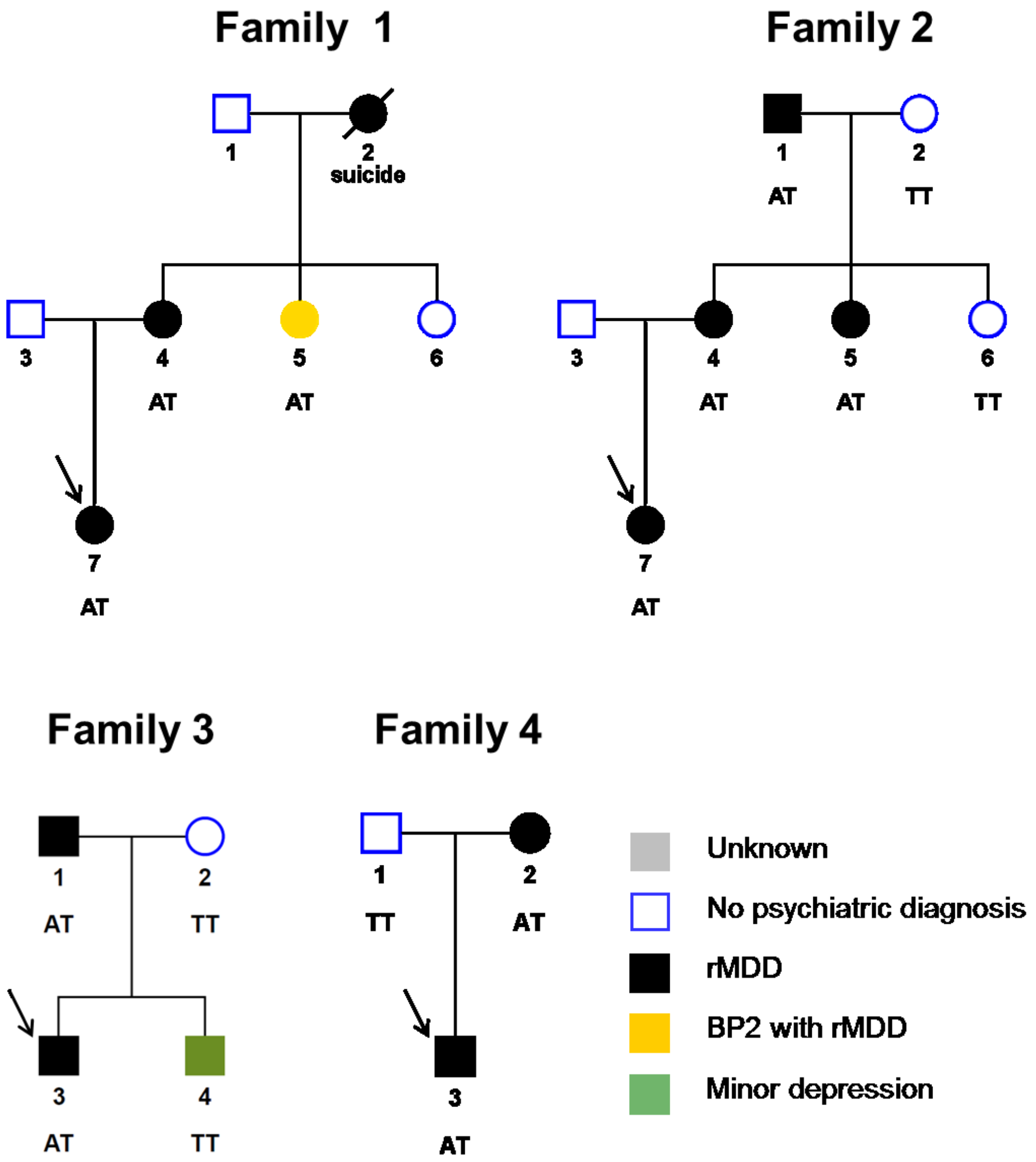

Additional family members, including an additional individual with recurrent major depressive disorder (rMDD), were available for four affected individuals, identified through secondary hospital referral, carrying the A risk allele at rs16856199. The proband of each family is indicated (arrows). rMDD, recurrent major depressive disorder; BP2, bipolar II.

Supplementary Figure S5. No segregation of rs16856199 In Generation Scotland rMDD families.

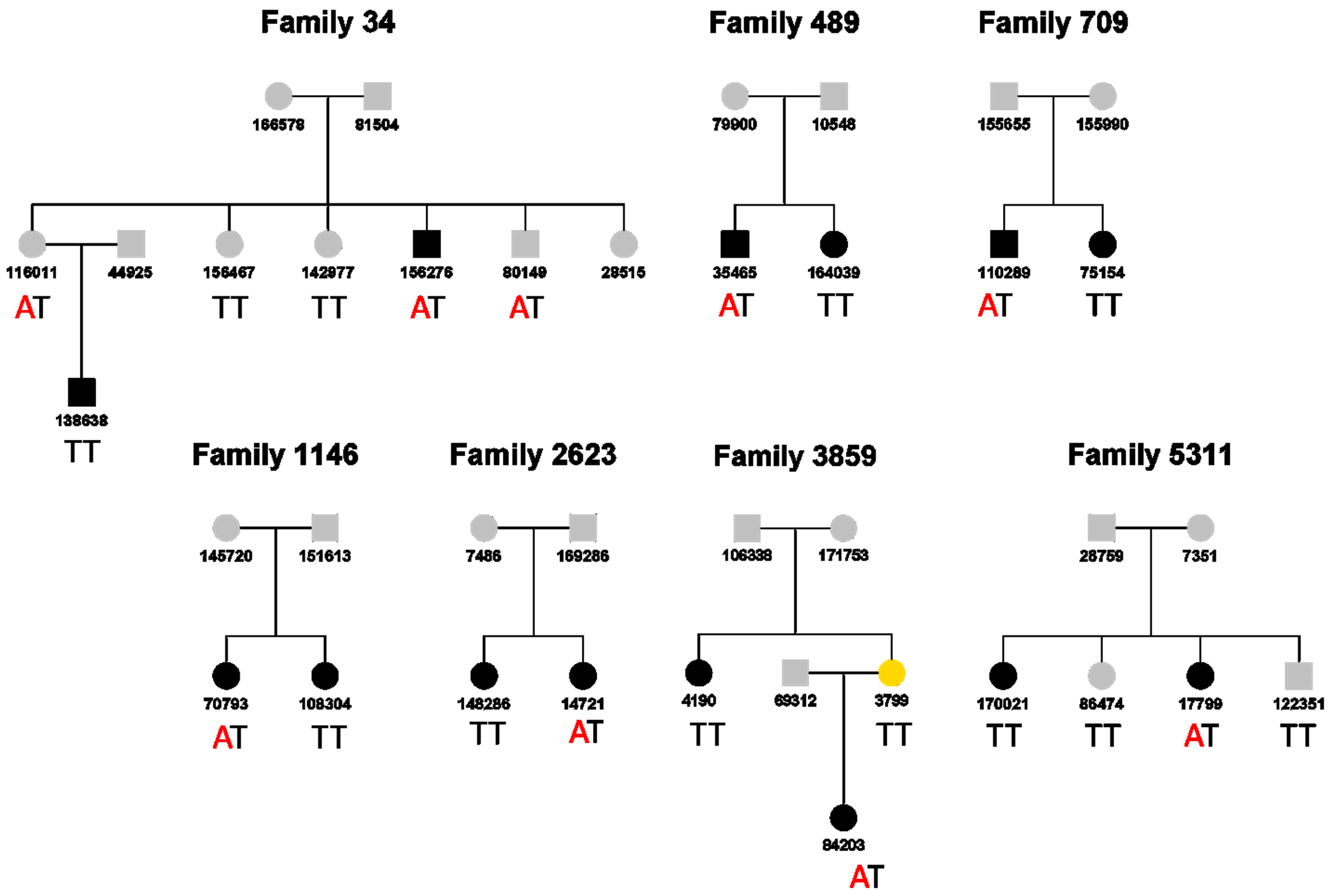

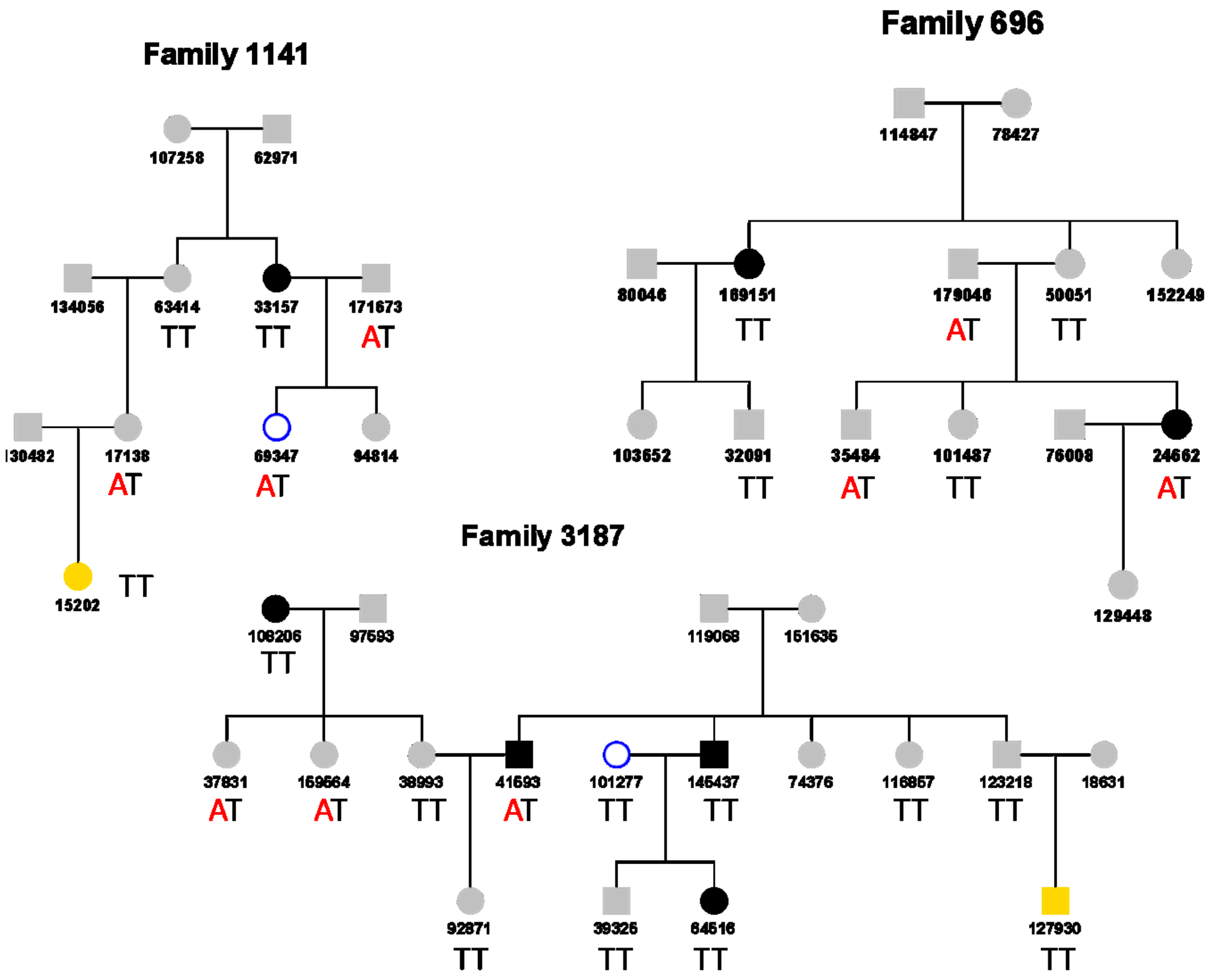
Family 1141
107258
62971
134056
63414
TT
33157
TT
171673
AT
130482
17138
AT
69347
AT
94814
15202
TT
Family 696
114847
78427
80046
169151
TT
179046
AT
50051
TT
152249
103652
32091
TT
35484
AT
101487
TT
76008
24662
AT
129448
Family 3187
108206
TT
97593
119068
151635
37831
AT
159564
AT
38993
TT
41693
AT
101277
TT
145437
TT
74376
116857
TT
123218
TT
18631
92871
TT
39326
TT
64516
TT
127930
TT

Supplementary Figure S6. Region-wide association analysis for cognitive traits in the LBC1936 controls.

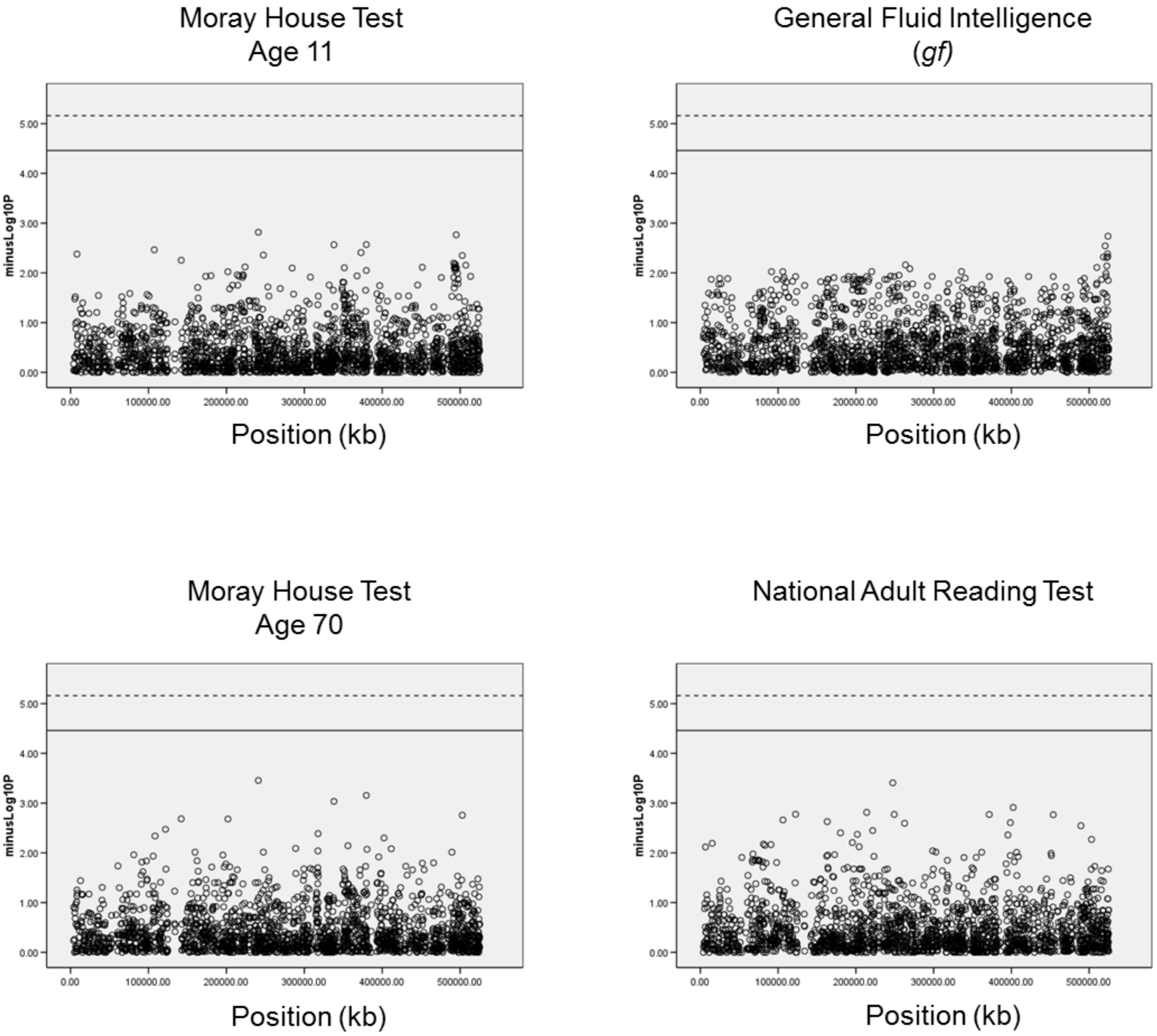

Empirical -log10(*P*-values) for linear regression analysis of each trait corrected for age at testing and sex are plotted against genomic location (hg18) across the *TRAX/DISC1* locus. Reference lines represent 1% (dashed) and 5% (solid) region-wide empirical thresholds. No SNP remained significant at <5%.

Supplementary Figure S7. Region-wide association analysis for cognitive-ageing traits in the LBC1936 controls.

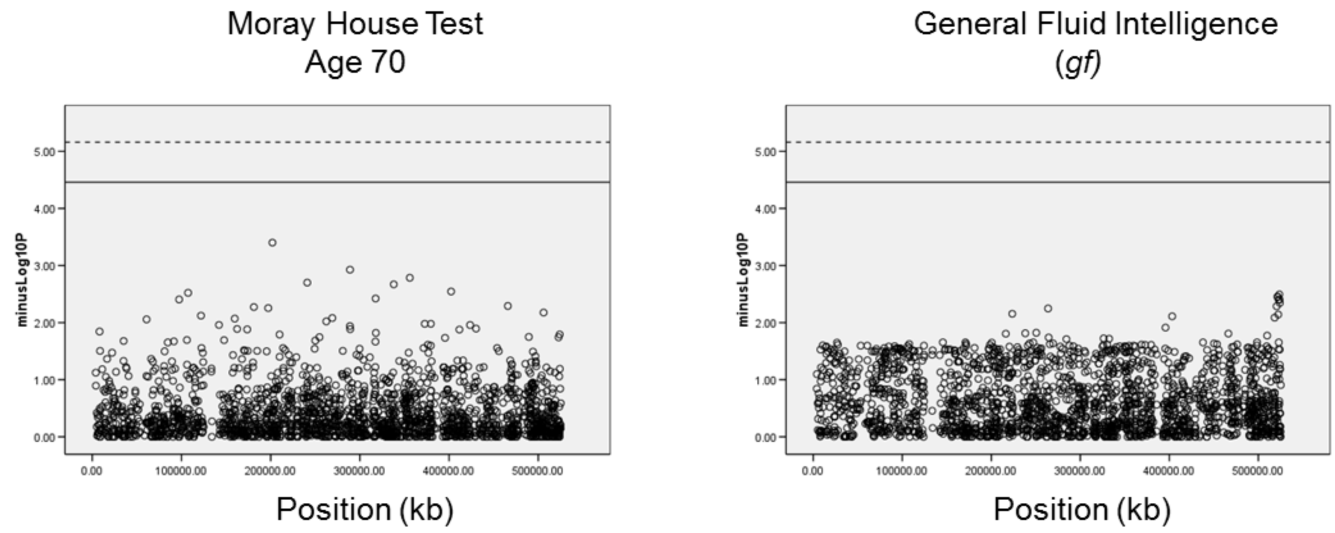

Empirical -log10(*P*-values) for linear regression analysis of each trait corrected for Moray House Test score at age 11, age at testing and sex are plotted against genomic location (hg18) across the *TRAX/DISC1* locus. Reference lines represent 1% (dashed) and 5% (solid) region-wide empirical thresholds. No SNP remained significant at <5%.

Supplementary Figure S8. Region-wide regression analysis for anxiety, neuroticism and depression in LBC1936.

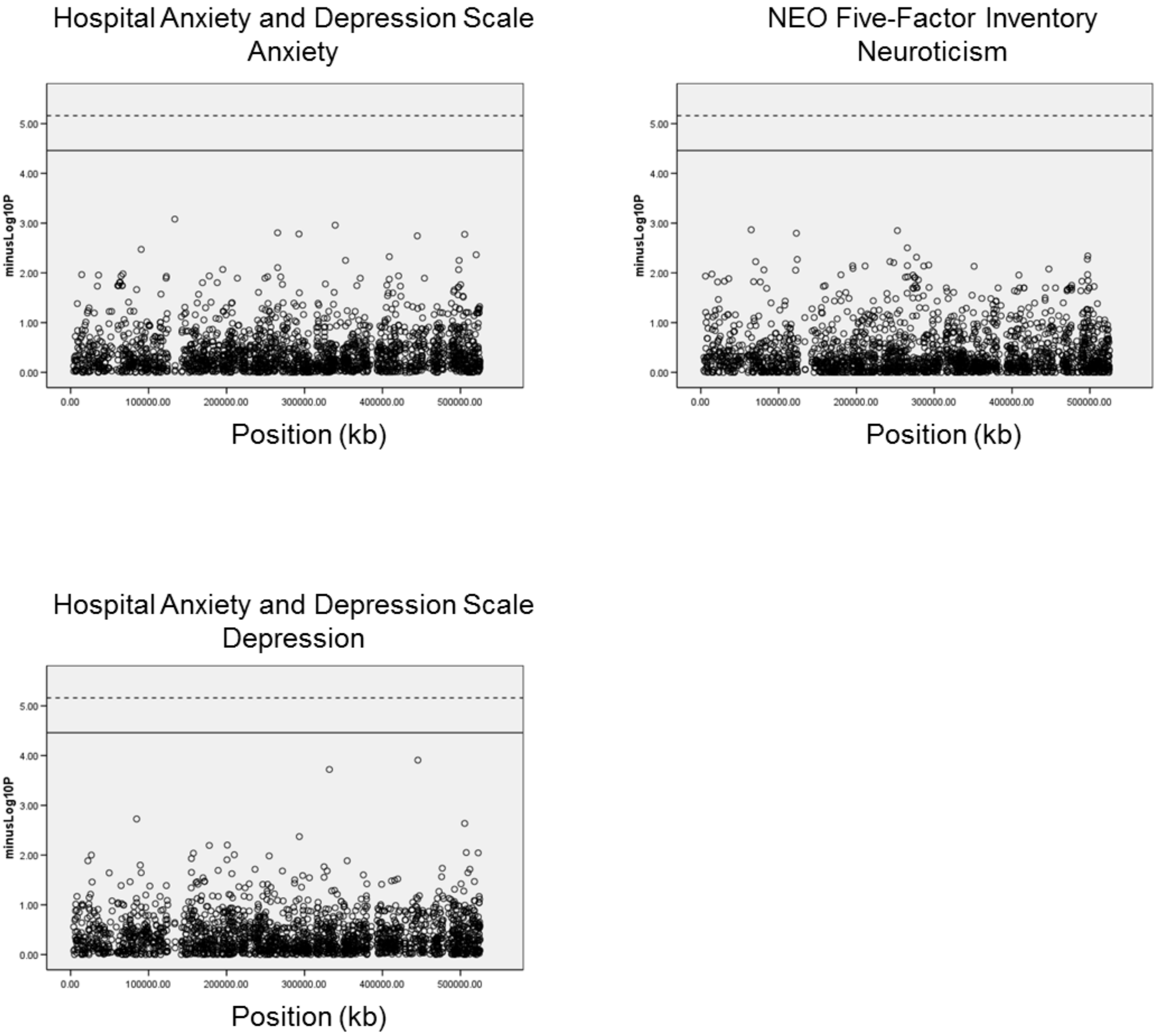

Empirical -log10(*P*-values) for linear regression analysis of each trait corrected for Moray House Test score at age 11, age at testing and sex are plotted against genomic location (hg18) across the *TRAX/DISC1* locus. Reference lines represent 1% (dashed) and 5% (solid) region-wide empirical thresholds. No SNP remained significant at <5%.

Supplementary Figure S9. Segregation of rare exonic SNPs.

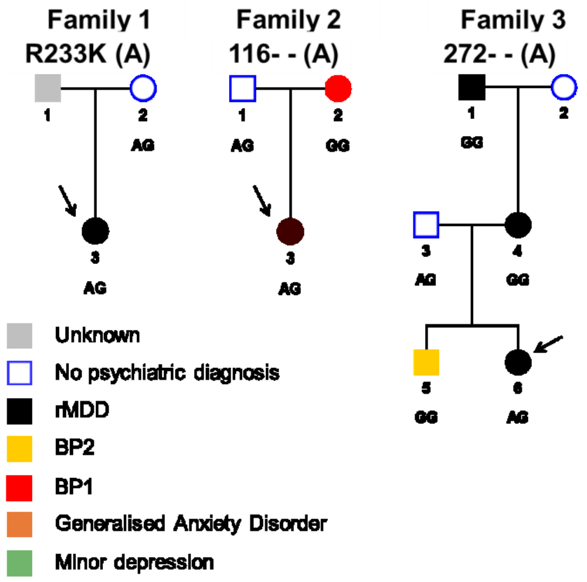

Family F224

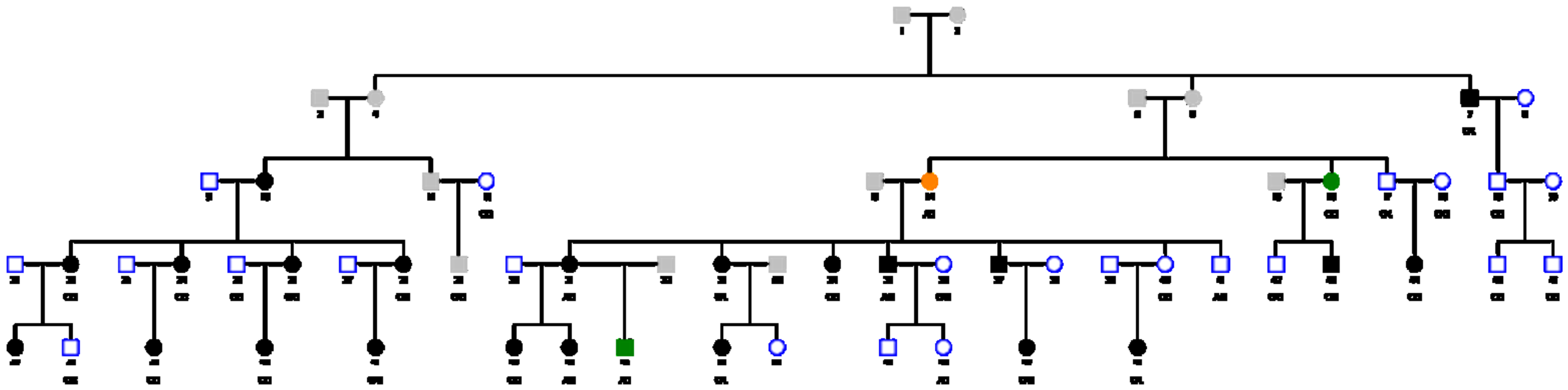

Supplementary Figure S10. No effect of diagnosis on number of singleton alleles per individual.

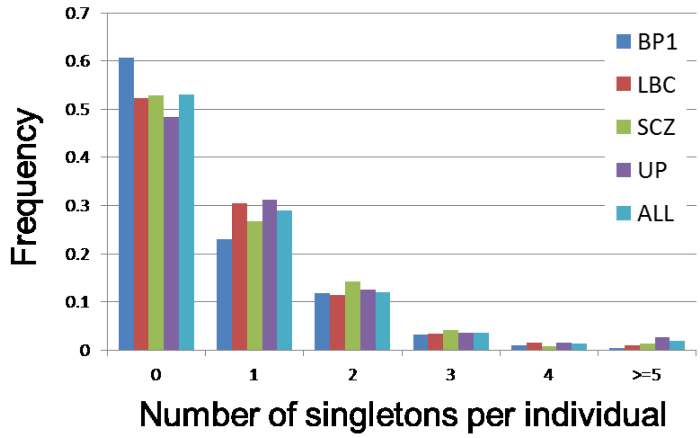

There was no significant difference between the number of novel alleles seen only once in the study per individual by diagnosis (p>0.05).

Supplementary Figure S11. Effect sizes detectable at 80% power for alpha=0.05

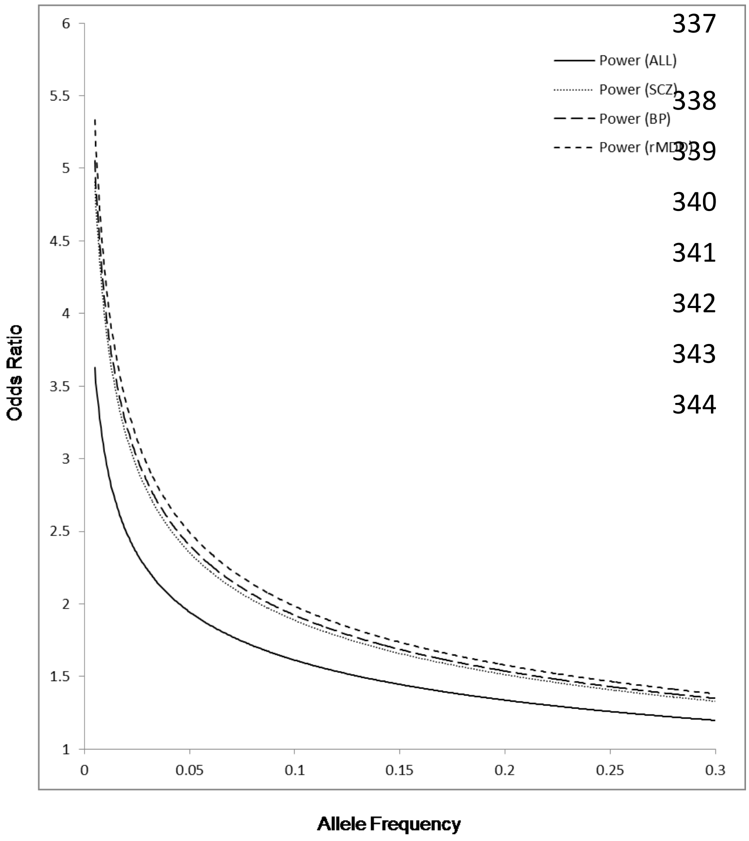

Effect size detectable with 80% power and a SNP alpha = 0.05 for the full study sample (ALL) and each of the study subsets: Schizophrenia – SCZ, Bipolar disorder – BP, Recurrent major depressive disorder - rMDD.

Supplementary Tables

Supplementary Table S1. Sequence analysis of the *TRAX/DISC1* locus: summary statistics.

Category	N	% Not in Repeats	% Validated	% Exonic Coding
**Number of Variants not in Repeats**	4243			
**After removal of tri-/quad – allelic and non-ref variants**	4099	96.6		
**After examination of Pile-up files, Validation and HWE**	2718	64.1		
**Remaining variants >= 0.01**	708		26.0	
**Remaining variants < 0.01**	2010		74.0	
**Number of known SNPs**	908		33.4	
**Number in EUR 1000 genomes (ALL)**	1027 (1221)		37.8	
**Number in regions of Regulatory Potential**	489		18.0	
**Number in Conversed regions (Phastcons)**	92		3.4	
**Number in Conserved TFBS**	19		0.7	
**Number in CpG Islands**	16		0.6	
**Number in Splice Sites**	3		0.1	
**Number in exons (excl DISC2)**	213 (145)		7.8	
**Number in Coding regions**	36		1.3	
**Synonymous Variants**	12			33.3
**Non-synonymous Variants**	23			63.9
**Predicted Stop Mutations**	1*			2.8

Seventy percent of SNPs identified in this study were not previously seen in Europeans, consistent with the high level of rare variation. Coding SNPs are annotated to the validated *TRAX* (otherwise known as *TSNAX*) isoform (UCSC gene: uc001huw.1) and DISC1 L isoform (UCSC gene: uc001huz.1). The single stop mutation corresponds to the short isoform of *DISC1* (UCSC gene: uc001huy.1).

Supplementary Table S2. Bioinformatic predictions of coding and regulatory variants in *TRAX/DISC1.*

See: DISC1_Seq_complexity__supp_table_S2.pdf

Supplementary Table S3. Overlap between variants identified in this study and *DISC1* candidate gene sequencing projects and relevant association studies.

See: DISC1_seq_complexity_supp_table_S3.pdf

Supplementary Table S4. Replication study of the association between rs1616856199 and recurrent major depressive disorder.

	Genotype Counts					Allele counts		Allele Freq				
	AA	AT	TT	Total	HWE p-value	A	T	%A	OR	95% CI low	95% CI high	*P*-value
Control	2	167	3848	4017	0.70	171	7863	2.13				
**CSH (DB1)**	2	16	156	174	0.096	20	328	5.75	3.48	1.95	6.23	**6.30E-05**
**Rep Only (DB2)**	1	26	440	467	0.34	28	906	3.00	1.42	0.95	2.13	0.088
**CombinedDB1&DB2**	3	41	594	639	0.048	47	1229	3.68	1.76	1.27	2.44	**0.00065**
MDD	1	30	659	690	0.31	32	1348	2.32	1.09	0.75	1.60	0.65
RMDD	1	30	614	645	0.33	32	1258	2.48	1.17	0.80	1.71	0.42
Combined GS	2	60	1273	1335	0.17	64	2606	2.40	1.13	0.84	1.51	0.41
**Combined GS&DB1&DB2**	5	101	1867	1974	0.018	111	3835	2.81	1.33	1.05	1.70	**0.02**
Combined GS&DB2	3	86	1713	1802	0.11	92	3512	2.55	1.21	0.93	1.56	0.15
**Combined rMDD DB1&DB2&GS**	4	71	1208	1284	0.029	79	2487	3.08	1.46	1.12	1.91	**0.0058**
Combined rMDD DB2&GS	2	56	1054	1112	0.19	60	2164	2.70	1.28	0.95	1.72	0.11

Original clinical rMDD samples	DB1
Replication clinical rMDD samples	DB2
Generation Scotland MDD +rMDD	Combined GS

In addition to the original clinical cohort (DB1), three samples were available for replication: additional secondary referrals from hospital outpatient clinics (DB2), a population-based sample of rMDD drawn from primary care as part of the Generation Scotland: Scottish Family Health Study (GS rMDD) and a third sample of 690 single episode MDD also drawn from Generation Scotland (GS MDD). These samples were analysed singularly and in combination against Generation Scotland screened controls.

Supplementary Table S5. Effect of limiting read depth on detection of singleton base changes.

	**Read depth**	**Singletons**		**Others**	
**Our Sequencing**	≥ 30	1121		1585	
		N missed if reads limited	%	N missed if reads limited	%
**Limited Reads**	1	572	51.0	122	7.7
	2	306	27.3	29	1.8
	3	154	13.7	6	0.4
	4	84	7.5	1	0.1
	5	53	4.7	0	0.0
	10	2	0.2	0	0.0
	20	0	0.0	0	0.0
	30	0	0.0	0	0.0

In this study genotypes were only accepted where the read depth was greater than or equal to 30 fold. To look for possible systematic differences in the ability to detect singletons (alleles seen only once in the study) with a reduced read depth, such as the read depth used in the 1000 Genomes Project pilot study, we reduced the reads taken for each of the study singletons and calculated if the SNP would have been detected at that reduced read depth.

Supplementary Table S6. Mark-Recapture estimation of TRAX/DISC1 common and rare variants in the European population.

					Predicted number of SNPs in the region					
	**CSHL**	**1000g EUR**	**Overlap**	**CSHL + 1000 g**	**Lincoln-Petersen**	**Modified Petersen**	**Var(N)**	**±95%CI**	**L**	**U**
All	2718	1515	1027	3206	4009.5	4008.7	3122.0	109.8	3898.9	4118.5
>1%	708	885	692	901	905.5	905.5	5.8	4.7	900.7	910.2
<1%	2010	630	335	2305	3780.0	3775.6	16383.0	251.5	3524.1	4027.1
Variables	C	M	R		MC/R					

Using the SNPs detected in the *TRAX/DISC1* region from whole genome sequencing of the European subset of the 1000 Genomes Project and those detected in this project, it is possible to estimate the total number of variants in the European population using the Lincoln-Petersen and Modified Petersen methods applied to mark-recapture experiments. 95% confidence intervals of this estimate were calculated following Chapman (1951). The total number of SNPs was estimated for all SNPs (All) and those found at >1% in this study (CSHL) or <1% in this study (CSHL).

<u>Lincoln-Petersen Method:</u>

$$N = \frac{MC}{R},$$

Where:
N = Estimate of total population size
M = Total number of variants identified in the first sample

C = Total number of variants identified in the second sample
R = Number of variants identified in the first sample that were then identified in the second sample

Modified Petersen Method:

$$N = \frac{(M+1)(C+1)}{R+1} - 1,$$

Where the variables are as above, and an approximately unbiased variance of N, or var(N), can be estimated by:

$$\mathrm{var}(N) = \frac{(M+1)(C+1)(M-R)(C-R)}{(R+1)(R+1)(R+2)}.$$

An approximate 95% CI (normality for N^c is assumed) can be estimated as:

$$N_c^{\wedge} \pm 1.965 * [var\ (N_c^{\wedge}\ )]^{0.5}$$

Supplementary Table S7. Classification of SNPs for burden analysis.

**Group**	**Track**	**Table**	**SNP subset for burden analysis**
Variation & repeats	Repeat masker	rsmk	
Genes/gene prediction tracks	UCSC genes	Known genes	Exonic, coding, non-synonymous
Variation & repeats	SNPs (135)	SNP 135	Known
Comparative genomics	Most conserved	PlacMammal	Conserved
Regulation	TBFS conserved	TF binding sites	TFBS
Regulation	7x potential	7x potential	Regulatory potential
Regulation	CpG	CpG	CpG

Supplementary Table S8: Case-Control rare variant burden analysis

BURDEN: burden of minor alleles (regardless of frequency)

Trait	All	Exonic	Coding	NS	PCon	RegPot	TFBS	CpG
Combined	0.170	0.604	0.916	0.725	0.374	0.146	0.788	0.267
SCZ	0.578	0.807	0.848	0.611	0.806	0.602	0.826	0.316
BP	0.242	0.717	0.933	0.742	0.159	0.128	0.463	0.328
rMDD	0.082	0.102	0.579	0.588	0.263	**0.044**	0.831	0.363

VTTEST: burden of minor alleles using an optimal minor allele frequency cutoff

Trait	All	Exonic	Coding	NS	PCon	RegPot	TFBS	CpG
Combined	0.331	0.995	0.932	0.854	0.159	0.465	0.781	0.517
SCZ	0.849	0.999	0.984	0.910	0.819	0.633	0.966	0.690
BP	0.939	0.956	0.973	0.505	0.431	0.167	0.538	0.264
rMDD	0.106	0.610	0.420	0.550	**0.022**	0.131	0.106	0.146

Pooled association of minor alleles was performed using both BURDEN,[16] which tests for excess of minor alleles in cases without any frequency cut-off, and VTTEST[17] which tests for excess of minor alleles in cases with an allele frequency cut-off optimally chosen (see supplementary methods). The *P*-values are shown for all SNPs (All) and SNPs annotated as potentially functional: Exonic, SNPs within coding and non-coding exons including the 5' and 3' UTRs; Coding, SNPs in protein-coding regions of exons including both synonymous and non-synonymous SNPs; NS, non-synonymous SNPs only; PCon, SNPs within regions of conservation in placental mammals (UCSC); RegPot, SNPs with putative regulatory potential (UCSC, 7 x regulatory potential); TFBS, SNPs in conserved transcription factor binding sites (UCSC); CpG, SNPs in CpG islands commonly associated with promoter regions.

Supplementary Table S9: Quantitative trait rare variant burden analysis

BURDEN: burden of minor alleles (regardless of frequency)

Trait	All	Exonic	Coding	NS	PCon	RegPot	TFBS	CpG
[a]General cognitive ability	0.551	0.087	0.830	0.813	0.410	0.630	0.134	0.352
General cognitive ability adjusted for Moray House Test at age 11*	0.238	0.096	0.558	0.713	0.171	0.212	0.121	0.809
[b]Symptoms of anxiety	0.509	0.719	0.724	0.712	0.420	0.437	0.224	0.216
[b]Symptoms of depression	0.699	0.355	0.449	0.785	0.261	0.544	**0.032**	0.664
[c]Moray House Test at age 70	0.707	0.652	0.906	0.920	0.695	0.626	0.149	0.295
Moray House Test at age 70 adjusted for the Moray house Test score at age 11	0.172	0.678	0.512	0.783	0.244	0.079	0.102	0.772
Moray House Test at age 11	0.935	0.414	0.934	0.830	0.876	0.961	0.561	**0.046**
[d]National Adult Reading Test	0.798	0.342	0.967	0.842	0.857	0.876	0.363	0.154
[e]Neuroticism	0.312	0.661	0.451	0.270	0.292	0.223	0.590	0.764

VTTEST: burden of minor alleles using an optimal minor allele frequency cutoff

Trait	All	Exonic	Coding	NS	PCon	RegPot	TFBS	CpG
[a]General cognitive ability	0.509	0.526	0.263	0.188	0.247	0.265	0.572	0.346
General cognitive ability adjusted for Moray House Test at age 11*	0.538	0.745	0.959	0.379	0.075	0.138	0.508	0.227
[b]Symptoms of anxiety	0.627	0.707	0.612	0.429	0.166	0.871	0.268	0.609
[b]Symptoms of depression	0.435	0.213	0.636	0.509	0.523	0.787	0.249	0.701

**[c]Moray House Test at age 70**	0.556	0.425	0.186	0.200	**0.030**	0.160	0.245	0.066
**Moray House Test at age 70 adjusted for the Moray House Test score at age 11**	0.527	0.922	0.422	0.393	0.164	0.095	0.111	**0.047**
**Moray House Test at age 11**	0.305	0.157	**0.028**	0.056	0.134	0.288	0.732	**0.040**
**[d]National Adult Reading Test**	0.281	0.607	0.251	0.405	0.081	0.139	0.072	0.124
**[e]Neuroticism**	0.657	0.783	0.790	0.640	0.620	0.786	0.544	0.212

Pooled association of minor alleles was performed using both BURDEN[16] which tests for excess of minor alleles in cases without any frequency cut-off, and VTTEST.[17] which tests for excess of minor alleles in cases with an allele frequency cut-off optimally chosen. Quantitative trait analysis in LBC1936 was one-tailed under the hypothesis that an increased burden of minor alleles would reduce scores for cognitive traits and increase scores for anxiety, depression and neuroticism. The *P*-values are shown for all SNPs (All) and SNPs annotated as potentially functional: Exonic, SNPs within coding and non-coding exons including the 5' and 3' UTRs; Coding, SNPs in protein-coding regions of exons including both synonymous and non-synonymous SNPs; NS, non-synonymous SNPs only; PCon, SNPs within regions of conservation in placental mammals (UCSC); RegPot, SNPs with putative regulatory potential (UCSC, 7 x regulatory potential); TFBS, SNPs in conserved transcription factor binding sites (UCSC); CpG, SNPs in CpG islands commonly associated with promoter regions. [a]This is 'fluid general cognitive ability' and is a combination (regression-based scores of a first unrotated principal component) of six subtests from the Wechsler Adult Intelligence Test-IIIUK: Matrix Reasoning, Block Design, Symbol Search, Digit Symbol, Letter-Number Sequencing, Backward Digit Span. [b]Measured using the Hospital Anxiety and Depressions Scales. [c]The Moray House Test is the general cognitive test—mostly a verbal reasoning, IQ-type test—that was used in the Scottish Mental Survey 1947. [d]This assesses 'crystallised' cognitive ability. [e]Measured using the NEO-Five Factor Inventory. References for all tests used in this table are available in the open-access Lothian Birth Cohort protocol paper (Deary et al., 2007).
