# Supplementary material for: 708 Common and 2010 rare DISC1 locus variants identified in 1542 subjects: analysis for association with psychiatric disorder and cognitive traits
Source: Mol Psychiatry. 2013 Jun 4;19(6):668–75. doi: 10.1038/mp.2013.68 (PMC4031635; doi:10.1038/mp.2013.68)
Supplement: Supplementary Table [file mp201368x2.pdf]

UCSC gene	Exon	SNP	ucscPos (hg18)	aa position	peptide shift	ref_ Base	Alt_ Base	SCZ	BP	rMDD	CTL	Case_MAF	CTL_ MAF	dbSNP132	1000genomes (hg19)	Eur1000 genomes MAF
TSNAX	Ex2	S008636	229731657	17	N|S	A	G	0/2/232	0/1/215	0/1/188	0/9/870	0.00309	0.00512		231665034	0.010
TSNAX	Ex2	S008640	229731661	18	-	C	T	0/1/233	0/0/216	0/0/189	0/3/876	0.00077	0.00171			
TSNAX	Ex3	S016607	229739628	56	-	T	C	0/0/241	0/0/221	0/0/192	**0/1/887**	0.00000	0.00056			
DISC1 (L)	Ex2	S173180	229896201	25	R|Q	G	A	0/1/235	0/2/213	0/1/188	0/2/871	0.00308	0.00115		231829578	0.000
DISC1 (L)	Ex2	S173215	229896236	37	R|W	C	T	0/0/232	0/0/211	**0/1/179**	0/0/847	0.00079	0.00000			
DISC1 (L)	Ex2	S173354	229896375	83	A|V	C	T	0/0/236	**0/1/215**	0/0/188	0/0/860	0.00077	0.00000			
DISC1 (L)	Ex2	S173375	229896396	90	S|L	C	T	0/0/236	0/0/216	0/0/190	**0/1/857**	0.00000	0.00058			
DISC1 (L)	Ex2	S173454	229896475	116	-	G	A	0/0/237	0/0/216	**0/2/187**	0/0/860	0.00154	0.00000			
DISC1 (L)	Ex2	S173563	229896584	153	S|P	T	C	0/0/237	0/0/216	0/0/189	**0/1/857**	0.00000	0.00058			
DISC1 (L)	Ex2	S173585	229896606	160	W|L	G	T	**0/1/235**	0/0/214	0/0/187	0/0/858	0.00077	0.00000			
DISC1 (L)	Ex2	S173700	229896721	198	-	C	A	0/0/236	0/0/216	0/0/190	**0/1/860**	0.00000	0.00058			
DISC1 (L)	Ex2	S173747	229896768	214	R|Q	G	A	0/0/236	0/0/216	0/0/188	**0/1/862**	0.00000	0.00058			
DISC1 (L)	Ex2	S173804	229896825	233	R|K	G	A	0/0/236	0/0/216	**0/1/190**	0/0/860	0.00077	0.00000			
DISC1 (L)	Ex2	S173816	229896837	237	S|Y	C	A	0/0/236	0/0/216	0/0/188	**0/1/858**	0.00000	0.00058			
DISC1 (L)	Ex2	S173897	229896918	264	R|Q	G	A	24/94/112	28/86/98	21/84/79	94/354/413	0.32830	0.31480	rs3738401	231830295	0.340
DISC1 (L)	Ex2	S173922	229896943	272	-	G	A	0/0/236	0/0/216	**0/1/188**	0/0/858	0.00077	0.00000			
DISC1 (L)	Ex2	S173966	229896987	287	P|L	C	T	0/0/237	0/1/215	0/0/188	0/1/864	0.00077	0.00058			
DISC1 (L)	Ex2	S173971	229896992	289	R|C	C	T	0/0/237	0/0/216	0/0/190	**0/1/866**	0.00000	0.00058			
DISC1 (L)	Ex4	S229284	229952305	376	-	A	G	0/0/240	0/0/217	**0/1/187**	0/0/884	0.00078	0.00000			
DISC1 (L)	Ex4	S229406	229952427	417	R|H	G	A	0/0/237	0/0/213	0/0/180	**0/1/858**	0.00000	0.00058			
DISC1 (L)	Ex4	S229409	229952430	418	R|H	G	A	**0/1/236**	0/0/215	0/0/181	0/0/861	0.00078	0.00000			
DISC1 (L)	Ex5	S246514	229969535	432	P|L	C	T	0/2/233	0/3/210	0/0/184	0/6/854	0.00390	0.00349	rs78792190		
DISC1 (L)	Ex5	S246577	229969598	453	T|M	C	T	0/0/236	0/3/210	0/2/182	0/11/842	0.00389	0.00645	rs28930675		
DISC1 (L)	Ex5	S246612	229969633	465	-	C	T	0/9/227	0/14/199	0/13/171	2/43/816	0.02804	0.02729	rs3738402	231903010	0.030
DISC1 (L)	Ex6	S250191	229973212	469	-	C	T	2/53/185	3/40/176	0/41/151	15/195/672	0.11140	0.12760	rs2492367	231906589	0.120
DISC1 (L)	Ex8	S279488	230002509	574	-	G	A	0/0/199	0/0/166	**0/1/153**	0/0/632	0.00095	0.00000			
DISC1 (L)	Ex9	S297692	230020713	603	T|M	C	T	0/0/218	0/0/186	0/0/159	**0/1/760**	0.00000	0.00066		231954090	0.000
DISC1 (L)	Ex9	S297703	230020724	607	L|F	C	T	8/46/151	4/39/130	3/25/123	19/179/554	0.13150	0.14430	rs6675281	231954101	0.110
DISC1 (L)	Ex9	S297747	230020768	621	-	G	A	0/19/200	0/14/173	1/20/137	3/78/682	0.04808	0.05505	rs12133766	231954145	0.050
DISC1 (L)	Ex11	S488200	230211221	704	S|C	A	T	18/80/120	15/57/125	12/59/97	66/300/451	0.24580	0.26440	rs821616	232144598	0.260
DISC1 (L) only	Ex11	S488341	230211362	751	E|Q	G	C	0/2/228	0/3/207	0/11/169	0/21/843	0.01274	0.01215	rs115112816	232144739	0.010
DISC1 (L)	Ex12	S505845	230228866	790	-	G	A	0/0/238	0/0/216	0/0/189	**0/1/888**	0.00000	0.00056			
DISC1 (L)	Ex13	S516116	230239137	834	-	G	A	0/0/236	0/0/209	0/0/173	**0/6/793**	0.00000	0.00376	rs41271517		

Known binding regions and Other notes adapted and updated from Soares et al. 2011.
Sequence conservation information inferred on the basis of the DISC1 orthologue multiple sequence alignment in Chubb et al. 2008;
hs: Homo sapiens, pt: Pan troglodytes, ma: Macaca mulatta , bt: Bos taurus, cf: Canis familiaris , rn: Rattus norvegicus, mo: Mus musculus, tr: Takifugu rubripes, dr: Danio rerio.
Predicted location on secondary structure based upon annotations made using methods described in Soares et al. 2011.

SNP	Pmut NN output / Prediction /Reliability	Panther SubPSEC / Pdeleterious	PolyPhen Prediction / PSIC difference	Variant resides within known interactor binding region	Other notes, functional motifs, experimental observations	Sequence conservation and location on predicted secondary structure
S008636	0.2331 / NEUTRAL / 5	-2.74464 / 0.43651	Benign / 0.004		Change to Ser not predicted to be phosphorylated by NetPhos	hs, pt, ma, rn; present as T in mo. Disordered loop (not seen in solved crystal structure).
S008640						
S016607						
S173180	0.4991 / NEUTRAL / 0	-1.34217 / 0.16005	Possibly damaging / 0.592	APP, eIF3 p40, KIF5A, MAP1A, PCM1, TRIO	Lies close to PDE4B binding site at residues 31-65.	hs, pt, ma; present as Q in cf. Disordered (Loop)
S173215	0.9622 / PATHOLOGICAL / 9	-5.1194 / 0.89277	Probably damaging / 1	APP, eIF3 p40, KIF5A, MAP1A, PCM1, PDE4B, TRIO	Refer main text.	hs, pt, ma, cf, rn, mo, tr, dr; Strictly conserved. Alpha-helix
S173354	0.5885 / PATHOLOGICAL / 1	-2.10681 / 0.29045	Possibly damaging / 0.903	APP, eIF3 p40, Kal-7, KIF5A, MAP1A, PCM1, TRIO		hs, pt, cf, rn, mo. Disordered (Loop)
S173375	0.1562 / NEUTRAL / 6	-1.09206 / 0.12921	Benign / 0	APP, eIF3 p40, Kal-7, KIF5A, MAP1A, PCM1, TRIO		hs, pt, cf. Disordered (Loop)
S173454						
S173563	0.6371 / PATHOLOGICAL / 2	-1.98336 / 0.26568	Probably damaging / 0.964	APP, eIF3 p40, KIF5A, MAP1A, PCM1, TRIO	Adjacent to strictly conserved residue S152 that is a predicted phosphorylation site by NetPhos.	hs, pt, ma, cf, rn. Disordered (Loop)
S173585	0.5798 / PATHOLOGICAL / 1	-0.84728 / 0.10408	Benign / 0.002	APP, eIF3 p40, KIF5A, MAP1A, PCM1, TRIO	Lies close to PDE4B binding site at residues 31-65.	hs, pt, ma; present as L in rn, mo. Disordered (Loop)
S173700						
S173747	0.6696 / PATHOLOGICAL / 3	-1.0072 / 0.11996	Benign / 0.25	APP, α-tubulin, eIF3 p40, GSK3β, KIF5A, MAP1A, Mitofilin, PCM1, PDE4 (general), TRIO	Located within conserved SF-rich region and proximal residue S216 is a predicted phosphorylation site by NetPhos.	hs, pt, ma, cf, rn; present as Q in mo, tr, dr. Alpha-helix
S173804	0.1171 / NEUTRAL / 7	-1.84379 / 0.23936	Probably damaging / 0.987	APP, α-tubulin, KIF5A, MAP1A, Mitofilin, PCM1, TRIO	Adjacent residue S232 is a predicted phosphorylation site by NetPhos. Close to: GSK3β binding site at 211-225, PDE4 (general) binding site at 191-230, and eIF3 p40 binding site at 1-231.	hs, pt, ma, cf, rn, mo, dr. Disordered (Loop)
S173816	0.2996 / NEUTRAL / 4	-4.25904 / 0.77886	Possibly damaging / 0.677	APP, α-tubulin, KIF5A, MAP1A, Mitofilin, PCM1, TRIO	Lies close to PDE4 (general) binding site at 191-230.	hs, pt, ma. Disordered (Loop)
S173897	0.5207 / PATHOLOGICAL / 0	-1.23049 / 0.1456	Benign / 0.033	APP, α-tubulin, KIF5A, MAP1A, Mitofilin, PCM1, TRIO	Lies close to peptide region in DISC1 266-290 mapped as PDE4B1 binding.	R in hs, pt, ma; Q not seen in any orthologue. Loop
S173922						
S173966	0.3341 / NEUTRAL / 3	-3.13082 / 0.53266	Benign / 0.005	APP, α-tubulin, KIF5A, MAP1A, Mitofilin, PCM1, PDE4B1, TRIO		hs, pt, ma, cf, rn, mo, tr. Disordered (Loop)
S173971	0.2366 / NEUTRAL / 5	-1.06244 / 0.12592	Benign / 0.002	APP, α-tubulin, KIF5A, MAP1A, Mitofilin, PCM1, PDE4B1, TRIO		hs, pt; present as C in ma, cf, rn, mo, tr. Disordered (Loop)
S229284						
S229406	0.5668 / PATHOLOGICAL / 1	-1.16204 / 0.13729	Benign / 0.01	ATF5, BBS1, BBS4, BBS8, Dysbindin, DISC1 (self-association), Girdin, GSK3β, MIP-T3	Also located near Kal-7 binding region (376-410).	hs, ma; present as H in rn, mo, dr. Alpha-helix
S229409	0.2171 / NEUTRAL / 5	-3.78157 / 0.68602	Benign / 0.037	ATF5, BBS1, BBS4, BBS8, Dysbindin, DISC1 (self-association), Girdin, GSK3β, MIP-T3	Also located near Kal-7 binding region (376-410).	hs, pt, ma, tr, dr; present as H in cf. End of regular alpha-helix
S246514	0.8459 / PATHOLOGICAL / 6	-2.14231 / 0.29782	Benign / 0.111	ATF5, BBS1, BBS4, BBS8, Dysbindin, DISC1 (self-association), Girdin, GSK3β, MIP-T3	Near predicted phosphorylation sites T429 and T431.	hs, rn, mo. Loop
S246577	0.8681 / PATHOLOGICAL / 7	-3.35029 / 0.58669	Probably damaging / 1	ATF5, BBS1, BBS4, BBS8, CAMDI, DBZ, Dysbindin, DISC1 (self-association), FEZ1, Girdin, GSK3β, Kendrin, MIP-T3		hs, pt, ma, bt, cf, rn, mo. Coiled coil helix.
S246612						
S250191						
S279488						
S297692	0.8738 / PATHOLOGICAL / 7	-3.83289 / 0.69697	Probably damaging / 0.972	ATF5, Dixdc1, FEZ1, N-CoR, PCM1	Predicted phosphorylation site by NetPhos. Also close to ATF4 binding region 606-628 and near PDE4 (general) binding region 611-650.	hs, pt, ma, bt, cf, rn, mo; present as S in tr, dr. Coiled coil helix
S297703	0.6076 / PATHOLOGICAL / 2	-3.5214 / 0.62747	Probably damaging / 1	ATF4, ATF5, Dixdc1, FEZ1, N-CoR, PCM1	Refer main text.	hs, pt, ma, bt, cf, rn, mo, tr, dr; Strictly conserved. Coiled coil helix
S297747						
S488200	0.8985 / PATHOLOGICAL / 7	-3.78328 / 0.68639	Probably damaging / 0.98	DISC1 (oligomerisation), Dixdc1, Girdin, Kendrin, N-CoR, PCM1	Refer main text.	hs, pt, ma, bt, rn, mo; present as G in cf, tr, dr. Alpha-helix
S488341	0.0768 / NEUTRAL / 8	-3.48552 / 0.61905	Probably damaging / 0.981	Dixdc1, Girdin, Kendrin, LIS1, Mitofilin, N-CoR, PCM1		hs, pt, ma, bt, rn, mo, tr; present as Q in dr. Alpha-helix
S505845						
S516116						
