# Supplementary material for: 708 Common and 2010 rare DISC1 locus variants identified in 1542 subjects: analysis for association with psychiatric disorder and cognitive traits
Source: Mol Psychiatry. 2013 Jun 4;19(6):668–75. doi: 10.1038/mp.2013.68 (PMC4031635; doi:10.1038/mp.2013.68)
Supplement: Supplementary Information [file mp201368x3.pdf]

Sorted by Variant (see below for by Study)

hg18	hg19	Author	Diagnosis	dbSNP135	AA change	NT change	Position	SNP	Genotype Distribution				Minor Allele Frequency				dbSNP	Obs Allele	Ref Allele	Literature Interpretation
									BP1	SCZ	rMDD	CTL	BP1	SCZ	UP	CTL				
229798979	231732356	Hennah W and Porteous D (2009)	Increases DISC1 expression levels	rs1765778		A>G		S075958	51/102/65	46/118/75	41/91/57	171/403/31	0.4678899	0.4393305	0.457672	0.419482	rs1765778	A/G	G	Displayed significant association in all four HapMap populations[d], minor allele associated with decreased DISC1 expression.
229800310	231733687	Hennah W and Porteous D (2009)	Increases DISC1 expression levels	rs1655297		T>C		S077289	34/80/56	36/92/80	26/66/67	105/333/31	0.4352941	0.3942308	0.3710692	0.3629679	rs1655297	A/G	G	Displayed significant association in three of the four HapMap populations exception being the Japanese population[d], minor allele associated with decreased DISC1 expression.
229817264	231750641	Hennah W and Porteous D (2009)	Increases DISC1 expression levels	rs1025526		T>C		S094243	32/102/87	31/93/113	26/73/92	95/365/428	0.3755656	0.3270042	0.3272251	0.3125	rs1025526	A/G	G	Displayed significant association in three of the four HapMap populations exception being the Japanese population[d], minor allele associated with decreased DISC1 expression.
229821502	231754879	Hennah W and Porteous D (2009)	Increases DISC1 expression levels	rs6541280		C>G		S098481	39/93/62	38/91/93	28/63/55	116/355/32	0.4407216	0.3761261	0.4075342	0.366875	rs6541280	C/G	G	Displayed significant association in three of the four HapMap populations exception being the Japanese population[d], minor allele associated with decreased DISC1 expression.
229828963	231762340	Hennah W and Porteous D (2009)	Increases DISC1 expression levels	rs3738398		C>G		S105942	30/47/22	35/49/50	20/43/19	99/228/162	0.540404	0.4440299	0.5060976	0.4355828	rs3738398	C/G	G	Displayed significant association in three of the four HapMap populations exception being the Japanese population[d], minor allele associated with decreased DISC1 expression.
229829096	231762473	Crowley et al. (2012)	SCZ + CTL				5 upstream	S106075	0/1/88	0/2/123	0/1/81	0/4/484	0.005618	0.008	0.006098	0.0040984				
229829230	231762607	Moens et al. (2011)	SCZ + CTL	rs3738399	7G>A	G>A	5'UTR	S106209	1/1/65	0/0/99	0/0/68	2/1/414	0.0223881	0	0	0.0059952	rs3738399	A/G	G	Found in both Cases and Controls.
229829274	231762651	Song et al. (2008)	SCZ + CTL		A13G	C>G	Exon1													
229829274	231762651	Song et al. (2010)	Bipolar Spectrum Disorder[c] + CTL		A13G	C>G	Exon1													Occurred in both Cases and Control, not associated with bipolar spectrum disorder.
229829277	231762654	Song et al. (2008)	SCZ		G14A	G>C	Exon1													Identified in Cases only.
229829306	231762683	Song et al. (2010)	Bipolar Spectrum Disorder[c]		IVS1 + 3	A>T	Intron1													Ultra-rare variant observed is statistically significant, found in Cases and absent in Controls and Gene Pool[b.]
229833853	231767230	Hennah W and Porteous D (2009)	Increases DISC1 expression levels	rs823167													rs823167	C/T	C	Displayed significant association in three of the four HapMap populations exception being the Japanese population[d], minor allele associated with decreased DISC1 expression.
229879331	231812708	Carless et al. (2011)	Influences brain structure	rs16854756													rs16854756	C/T	T	
229896201	231829578	Song et al. (2010)	Bipolar Spectrum Disorder[c] + CTL	rs143922209	R25Q	G>A	Exon2	S173180	0/2/213	0/1/235	0/1/188	0/2/871	0.0046512	0.0021186	0.0026455	0.0011455				
229896201	231829578	Crowley et al. (2012)	SCZ + CTL	rs143922209	R25Q	G>A	Exon2	S173180	0/2/213	0/1/235	0/1/188	0/2/871	0.0046512	0.0021186	0.0026455	0.0011455				3' UTR
229896236	231829613	Song et al. (2008)	SCZ		R37W	C>T	Exon2	S173215	0/0/211	0/0/232	0/1/179	0/0/847	0	0	0.0027778	0				Identified in Cases only.
229896323	231829700	Crowley et al. (2012)	SCZ + CTL[e]																	Mis-sense
229896338	231829715	Osbun et al. (2011)	AgCC		V71L	G>T	Exon2													
229896338	231829715	Crowley et al. (2012)	SCZ																	Mis-sense, PolyPhen - benign, SIFT - tolerated
229896375	231829752	Song et al. (2008)	SCZ + CTL	rs76175896	A83V	C>T	Exon2	S173354	0/1/215	0/0/236	0/0/188	0/0/860	0.0023148	0	0	0				Identified in both Cases and Gene Pool[b.]
229896375	231829752	Osbun et al. (2011)	AgCC	rs76175896	A83V	C>T	Exon2	S173354	0/1/215	0/0/236	0/0/188	0/0/860	0.0023148	0	0	0				
229896375	231829752	Moens et al. (2011)	SCZ + CTL	rs76175896	A83V	C>T	Exon2	S173354	0/1/215	0/0/236	0/0/188	0/0/860	0.0023148	0	0	0	rs76175896			Found in both Cases and Controls, Possible splicing effect.
229896396	231829773	Song et al. (2008)	SCZ		S90L	C>T	Exon2	S173375	0/0/216	0/0/236	0/0/190	0/1/857	0	0	0	0.0005828				Identified in Cases only.
229896396	231829773	Crowley et al. (2012)	SCZ		S90L	C>T	Exon2	S173375	0/0/216	0/0/236	0/0/190	0/1/857	0	0	0	0.0005828				Mis-sense, PolyPhen - benign, SIFT - tolerated
229896606	231829983	Moens et al. (2011)	SCZ + CTL	rs143796295	W160L	G>T	Exon2	S173585	0/0/214	0/1/235	0/0/187	0/0/858	0	0.0021186	0	0				Found in both Cases and Controls, No obvious functional effect.
229896606	231829983	Crowley et al. (2012)	SCZ + CTL	rs143796295	W160L	G>T	Exon2	S173585	0/0/214	0/1/235	0/0/187	0/0/858	0	0.0021186	0	0				Mis-sense, PolyPhen - possibly damaging, SIFT - tolerated
229896754	231830131	Song et al. (2010)	Bipolar Spectrum Disorder[c]		S209R	C>G[a]	Exon2													Ultra-rare variant observed is statistically significant, found in Cases and absent in Controls and Gene Pool[b.]
229896917	231830294	Song et al. (2008)	CTL		Q264C	C>T	Exon2													Identified in both Cases and Gene Pool[b.]
229896917	231830294	Song et al. (2010)	CTL		Q264C	C>T	Exon2													
229896918	231830295	Song et al. (2008)	SCZ + CTL	rs3738401	Q264R	A>G	Exon2	S173897	28/86/98	24/94/112	21/84/79	94/354/413	0.3349057	0.3086957	0.3423913	0.3147503	rs3738401	A/G	G	Associated with elevating the risk for Schizophrenia.
229896918	231830295	Song et al. (2010)	Bipolar Spectrum Disorder[c] + CTL	rs3738401	Q264R	A>G	Exon2	S173897	28/86/98	24/94/112	21/84/79	94/354/413	0.3349057	0.3086957	0.3423913	0.3147503	rs3738401	A/G	G	Occurred in both Cases and Control, not associated with bipolar spectrum disorder.
229896918	231830295	Carless et al. (2011)	Influences brain structure	rs3738401	Q264R	G>A	Exon2	S173897	28/86/98	24/94/112	21/84/79	94/354/413	0.3349057	0.3086957	0.3423913	0.3147503	rs3738401	A/G	G	Observed moderate evidence for cis-regulation in significantly larger population size.
229896918	231830295	Osbun et al. (2011)	AgCC + CTL	rs3738401	R264Q	G>A	Exon2	S173897	28/86/98	24/94/112	21/84/79	94/354/413	0.3349057	0.3086957	0.3423913	0.3147503	rs3738401	A/G	G	Known to be associated with higher risk of developing SCZ, did not reach the statistical significance in this study.
229896918	231830295	Moens et al. (2011)	SCZ + CTL	rs3738401	R264Q	G>A	Exon2	S173897	28/86/98	24/94/112	21/84/79	94/354/413	0.3349057	0.3086957	0.3423913	0.3147503	rs3738401	A/G	G	Found in both Cases and Controls, Possible splicing effect.
229896918	231830295	Crowley et al. (2012)	SCZ + CTL	rs3738401	Q264R	A>G	Exon2	S173897	28/86/98	24/94/112	21/84/79	94/354/413	0.3349057	0.3086957	0.3423913	0.3147503	rs3738401	A/G	G	Mis-sense, PolyPhen - benign, SIFT - tolerated
229896948	231830325	Crowley et al. (2012)	SCZ																	Mis-sense, PolyPhen - benign, SIFT - tolerated
229896987	231830364	Song et al. (2010)	Bipolar Spectrum Disorder[c] + CTL	rs146244276	P287L	C>T	Exon2	S173966	0/1/215	0/0/237	0/0/188	0/1/864	0.0023148	0	0	0.000578				Occurred in both Cases and Control, not associated with bipolar spectrum disorder.
229896987	231830364	Osbun et al. (2011)	AgCC + CTL	rs146244276	P287L	C>T	Exon2	S173966	0/1/215	0/0/237	0/0/188	0/1/864	0.0023148	0	0	0.000578				Detected at low frequencies in both Cases and Control.
229896987	231830364	Crowley et al. (2012)	SCZ + CTL	rs146244276	P287L	C>T	Exon2	S173966	0/1/215	0/0/237	0/0/188	0/1/864	0.0023148	0	0	0.000578				Mis-sense, PolyPhen - benign, SIFT - tolerated
229897014	231830391	Song et al. (2010)	CTL		D296G	A>G	Exon2													
229897140	231830517	Song et al. (2010)	Bipolar Spectrum Disorder[c]		R338Q	G>A	Exon2													Ultra-rare variant observed is statistically significant, found in Cases and absent in Controls and Gene Pool[b.]
229924937	231858314	Crowley et al. (2012)	CTL		W359*	G>A	Intron3	S201916	0/0/207	0/0/233	0/0/186	0/1/756	0	0	0	0.0006605				Non-sense
229924986	231858363	Crowley et al. (2012)	CTL				Intron3	S201965	0/0/216	0/0/235	0/0/191	0/1/793	0	0	0	0.0006297				3' UTR
229925021	231858398	Crowley et al. (2012)	CTL																	3' UTR
229929827	231863204	Carless et al. (2011)	Influences brain structure	rs2487453		C>T	Intron3	S206806	35/87/87	30/102/101	26/88/68	115/381/37	0.3755981	0.3476395	0.3846154	0.350344	rs2487453	A/G	A	Found to be cis-acting and influences brain structure, specifically thickness of the cingulate cortex
229943605	231876982	Carless et al. (2011)	Spatial working memory	rs2793094													rs2793094	C/T	C	Associations were detected with measures of spatial working memory
229952259	231885636	Osbun et al. (2011)	AgCC		IVS4 – 36	T>C	Intron4													
229952430	231885807	Song et al. (2008)	SCZ	rs144959108	R418H	G>A	Exon4	S229409	0/0/215	0/1/236	0/0/181	0/0/861	0	0.0021097	0	0				Identified in Cases only.
229952430	231885807	Song et al. (2010)	Bipolar Spectrum Disorder[c]	rs144959108	R418H	G>A	Exon4	S229409	0/0/215	0/1/236	0/0/181	0/0/861	0	0.0021097	0	0				Identified as ultra-rare variant in SCZ study too (Song et al., 2008).
229963491	231896868	Hennah et al. (2009)	Interplay SNPs	rs1538979		C>T		S240470	1/38/175	4/46/183	6/41/134	25/201/645	0.0934579	0.1158798	0.1464088	0.1440873	rs1538979	A/G	G	Significant association observed in Finnish BP1 males, no significant odds ratios in combined cohorts.
229969535	231902912	Song et al. (2008)	SCZ + CTL	rs78792190	P432L	C>T	Exon5	S246514	0/3/210	0/2/233	0/0/184	0/6/854	0.0070423	0.0042553	0	0.0034884				Identified in both Cases and Gene Pool[b.]
229969535	231902912	Song et al. (2010)	Bipolar Spectrum Disorder[c] + CTL	rs78792190	P432L	C>T	Exon5	S246514	0/3/210	0/2/233	0/0/184	0/6/854	0.0070423	0.0042553	0	0.0034884				Occurred in both Cases and Control, not associated with bipolar spectrum disorder.
229969535	231902912	Crowley et al. (2012)	SCZ + CTL	rs78792190	P432L	C>T	Exon5	S246514	0/3/210	0/2/233	0/0/184	0/6/854	0.0070423	0.0042553	0	0.0034884				Mis-sense, PolyPhen - possibly damaging, SIFT - damaging
229969598	231902975	Song et al. (2008)	SCZ + CTL	rs28930675	T453M	C>T	Exon5	S246577	0/3/210	0/0/236	0/2/182	0/11/842	0.0070423	0	0.0054348	0.0064478	rs28930675	C/T	C	
229969598	231902975	Song et al. (2010)	Bipolar Spectrum Disorder[c] + CTL	rs28930675	T453M	C>T	Exon5	S246577	0/3/210	0/0/236	0/2/182	0/11/842	0.0070423	0	0.0054348	0.0064478	rs28930675	C/T	C	Occurred in both Cases and Control, not associated with bipolar spectrum disorder.
229969598	231902975	Osbun et al. (2011)	AgCC + CTL	rs28930675	T453M	C>T	Exon5	S246577	0/3/210	0/0/236	0/2/182	0/11/842	0.0070423	0	0.0054348	0.0064478	rs28930675	C/T	C	Detected at low frequencies in both Cases and Control.
229969633	231903010	Osbun et al. (2011)	AgCC	rs3738402	L465L	C>T	Exon5	S246612	0/14/199	0/9/227	0/13/171	2/43/816	0.0328638	0.0190678	0.0353261	0.0272938	rs3738402	C/T	C	
229969633	231903010	Moens et al. (2011)	SCZ + CTL	rs3738402	L465L	C>T	Exon5	S246612	0/14/199	0/9/227	0/13/171	2/43/816	0.0328638	0.0190678	0.0353261	0.0272938	rs3738402	C/T	C	Found in both Cases and Controls, Possible amino acid effect.
229973212	231906589	Osbun et al. (2011)	AgCC	rs2492367	I469I	C>T	Exon6	S250191	3/40/176	2/53/185	0/41/151	15/195/672	0.1050228	0.11875	0.1067708	0.127551	rs2492367	C/T	C	
229973212	231906589	Moens et al. (2011)	SCZ + CTL	rs2492367	I469I	C>T	Exon6	S250191	3/40/176	2/53/185	0/41/151	15/195/672	0.1050228	0.11875	0.1067708	0.127551	rs2492367	C/T	C	Found in both Cases and Controls, Possible splicing effect.
229973321	231906698	Crowley et al. (2012)	SCZ[e]																	Mis-sense, PolyPhen - possibly damaging, SIFT - tolerated
229973424	231906801	Song et al. (2010)	Bipolar Spectrum Disorder[c] + CTL	rs143165003	P540Q	C>A	Exon6													Occurred in both Cases and Control, not associated with bipolar spectrum disorder.
229973424	231906801	Osbun et al. (2011)	AgCC	rs143165003	P540Q	C>A	Exon6													Identified in Cases only.
229973424	231906801	Crowley et al. (2012)	SCZ	rs143165003	P540Q	C>A	Exon6													Mis-sense, PolyPhen - possibly damaging, SIFT - damaging
229973446	231906823	Crowley et al. (2012)	SCZ + CTL[e]																	Splice-region
229973470	231906847	Osbun et al. (2011)	AgCC	rs16854940	IVS6 + 31	C>T	Intron6	S250449	0/14/207	0/9/231	0/13/179	2/45/838	0.0316742	0.01875	0.0338542	0.0276836	rs16854940	C/T	C	
229979215	231912592	Carless et al. (2011)	Influences brain structure	rs16854954		T>C		S256194	24/72/112	25/90/112	16/76/93	71/342/423	0.2884615	0.30837	0.2918919	0.2894737	rs16854954	C/T	T	
229997522	231930899	Osbun et al. (2011)	AgCC	rs41271515	IVS6 – 89	G>A	Intron6	S274501	0/1/219	0/0/240	0/0/191	0/3/879	0.0022727	0	0	0.0017007	rs41271515	A/G	G	
229997670	231931047	Osbun et al. (2011)	AgCC		IVS7 + 6	T>C	Intron7													
229997671	231931048	Moens et al. (2011)	SCZ + CTL	rs2273890	169488T>C	T>C	Splice/Intron7	S274650	4/60/157	9/56/175	4/44/143	20/213/655	0.1538462	0.1541667	0.1361257	0.142455	rs2273890	A/G	A	Found in both Cases and Controls, Possible splicing effect (5' splice site).
230002487	231935864	Song et al. (2010)	Bipolar Spectrum Disorder[c] + CTL		S567N	G>A	Exon8													
230002543	231935920	Crowley et al. (2012)	SCZ																	Mis-sense, PolyPhen - possibly damaging, SIFT - tolerated
230002639	231936016	Osbun et al. (2011)	AgCC	rs2759354	IVS8 + 60	G>A	Intron8	S279618	0/2/207	0/2/233	0/3/183	0/13/867	0.0047847	0.0042553	0.0080645	0.0073864	rs2759354	C/T	T	
230020713	231954090	Song et al. (2008)	SCZ		T603I	C>T	Exon9													Identified in Cases only.
230020724	231954101	Song et al. (2008)	SCZ + CTL	rs6675281	L607F	C>T	Exon9	S297703	4/39/130	8/46/151	3/25/123	19/179/554	0.1358382	0.1512195	0.102649	0.1442819	rs6675281	C/T	C	Previously associated with elevating the risk for Schizophrenia, no evidence found.
230020724	231954101	Song et al. (2010)	Bipolar Spectrum Disorder[c] + CTL	rs6675281	L607F	C>T	Exon9	S297703	4/39/130	8/46/151	3/25/123	19/179/554	0.1358382	0.1512195	0.102649	0.1442819	rs6675281	C/T	C	Occurred in both Cases and Control, not associated with bipolar spectrum disorder.
230020724	231954101	Osbun et al. (2011)	AgCC + CTL	rs6675281	L607F	C>T	Exon9	S297703	4/39/130	8/46/151	3/25/123	19/179/554	0.1358382	0.1512195	0.102649	0.1442819	rs6675281	C/T	C	Known to be associated with higher risk of developing SCZ, did not reach the statistical significance in this study.
230020724	231954101	Moens et al. (2011)	SCZ + CTL	rs6675281	L607F	C>T	Exon9	S297703	4/39/130	8/46/151	3/25/123	19/179/554	0.1358382	0.1512195	0.102649	0.1442819	rs6675281	C/T	C	
230020724	231954101	Crowley et al. (2012)	SCZ + CTL	rs6675281	L607F	C>T	Exon9	S297703	4/39/130	8/46/151	3/25/123	19/179/554	0.1358382	0.1512195	0.102649	0.1442819	rs6675281	C/T	C	Mis-sense, PolyPhen - benign, SIFT - damaging
230020768	231954145	Osbun et al. (2011)	AgCC	rs12133766	L621L	G>A	Exon9	S297747	0/14/173	0/19/200	1/20/137	3/78/682	0.0374332	0.043379	0.0696203	0.0550459	rs12133766	A/G	G	
230020768	231954145	Moens et al. (2011)	SCZ + CTL	rs12133766	L621L	G>A	Exon9	S297747	0/14/173	0/19/200	1/20/137	3/78/682	0.0374332	0.043379	0.0696203	0.0550459	rs12133766	A/G	G	Found in both Cases and Controls, No obvious functional effect (though genomically conserved).
230069015	232002392	Moens et al. (2011)	SCZ + CTL	rs3082	2105A>G	A>G	3'UTR/Intron9										rs3082	C/T	T	
230081663	232015040	Carless et al. (2011)	Influences brain structure	rs2356606													rs2356606	C/T	T	Found to be associated with thickness of the entorhinal cortex and postcentral gyrus
230087871	232021248	Carless et al. (2011)	Influences brain structure	rs9431714		G>A		S364850	33/103/76	34/109/92	29/76/79	119/399/36	0.3985849	0.3765957	0.3641304	0.361521	rs9431714	A/G	G	
230133680	232067057	Hennah et al. (2009)	Interplay SNPs	rs821577		T>G		S410659	40/110/66	42/114/83	38/93/60	171/413/29	0.4398148	0.4142259	0.4424084	0.4289773	rs821577	G/T	T	Significant association observed in the London BP females, showed evidence for increased risk within the combined cohorts.
230145237	232078614	Carless et al. (2011)	Major depressive disorder	rs4658966		T>C		S422216	4/49/140	5/52/166	2/38/130	16/179/614	0.1476684	0.1390135	0.1235294	0.1304079	rs4658966	C/T	T	Associations were detected with recurrent major depression disorder
230152776	232086153	Carless et al. (2011)	Major depressive disorder + Lifetime panic	rs12137417													rs12137417	A/G	G	Associations were detected with recurrent major depression disorder and lifetime measures of panic
230161188	232094565	Crowley et al. (2012)	SCZ + CTL																	Splice-region
230161352	232094729	Osbun et al. (2011)	AgCC	rs2794273	IVS10 + 95	G>A	Intron10	S438331	9/61/103	14/78/105	11/55/80	45/253/341	0.2283237	0.2690355	0.2636986	0.2683881	rs2794273	C/T	C	
230211153	232144530	Osbun et al. (2011)	AgCC		IVS10 – 2	A>G	Intron10													Identified in Cases only.
230211174	232144551	Crowley et al. (2012)	SCZ[e]																	Mis-sense, PolyPhen - benign, SIFT - tolerated
230211221	232144598	Song et al. (2008)	SCZ + CTL	rs821616	S704C	A>T	Exon11	S488200	15/57/125	18/80/120	12/59/97	66/300/451	0.2208122	0.266055	0.2470238	0.2643819	rs821616	A/T	T	Associated with elevating the risk for Schizophrenia.
230211221	232144598	Song et al. (2010)	Bipolar Spectrum Disorder[c] + CTL	rs821616	C704S	A>T	Exon11	S488200	15/57/125	18/80/120	12/59/97	66/300/451	0.2208122	0.266055	0.2470238	0.2643819	rs821616	A/T	T	Occurred in both Cases and Control, not associated with bipolar spectrum disorder.
230211221	232144598	Osbun et al. (2011)	AgCC + CTL	rs821616	S704C	A>T	Exon11	S488200	15/57/125	18/80/120	12/59/97	66/300/451	0.2208122	0.266055	0.2470238	0.2643819	rs821616	A/T	T	Known to be associated with higher risk of developing SCZ, did not reach the statistical significance in this study.
230211221	232144598	Moens et al. (2011)	SCZ + CTL	rs821616	S704C	A>T	Exon11	S488200	15/57/125	18/80/120	12/59/97	66/300/451	0.2208122	0.266055	0.2470238	0.2643819	rs821616	A/T	T	Found in both Cases and Controls, Possible amino acid effect.
230211221	232144598	Crowley et al. (2012)	SCZ + CTL	rs821616	S704C	A>T	Exon11	S488200	15/57/125	18/80/120	12/59/97	66/300/451	0.2208122	0.266055	0.2470238	0.2643819	rs821616	A/T	T	Mis-sense, PolyPhen - unknown, SIFT - damaging
230211271	232144648	Green et al. (2011)	RDC – SABP		R720R	G>A	Exon11													Identified in Cases only.
230211319	232144696	Green et al. (2011)	RDC – SABP		L736L	C>T	Exon11													Identified in Cases only.
230211362	232144739	Song et al. (2008)	SCZ + CTL	rs115112816	E751Q	G>C	Exon11	S488341	0/3/207	0/2/228	0/11/169	0/21/843	0.0071429	0.0043478	0.0305556	0.0121528				
230211362	232144739	Song et al. (2010)	Bipolar Spectrum Disorder[c] + CTL	rs115112816	E751Q	G>C	Exon11	S488341	0/3/207	0/2/228	0/11/169	0/21/843	0.0071429	0.0043478	0.0305556	0.0121528				Occurred in both Cases and Control, not associated with bipolar spectrum disorder.
230211362	232144739	Green et al. (2011)	RDC - SABP + DSM-IV – RDC + CTL	rs115112816	E751Q	G>C	Exon11	S488341	0/3/207	0/2/228	0/11/169	0/21/843	0.0071429	0.0043478	0.0305556	0.0121528				Identified in both Cases and Controls.
230211362	232144739	Moens et al. (2011)	SCZ + CTL	rs115112816	E751Q	G>C	Exon11	S488341	0/3/207	0/2/228	0/11/169	0/21/843	0.0071429	0.0043478	0.0305556	0.0121528				Found in both Cases and Controls, No obvious functional effect (though genomically conserved).
230211362	232144739	Crowley et al. (2012)	SCZ + CTL	rs115112816	E751Q	G>C	Exon11	S488341	0/3/207	0/2/228	0/11/169	0/21/843	0.0071429	0.0043478	0.0305556	0.0121528				Mis-sense, PolyPhen - unknown, SIFT - damaging

**230211363**	232144740	Green et al. (2011)	RDC – SABP + DSM-IV – RDC +CTL	rs183265932	**E751A**	A>C	Exon11													Identified in both Cases and Controls.
230211372	232144749	Song et al. (2010)	Bipolar Spectrum Disorder[c]		**T754S**	C>G	Exon11													Ultra-rare variant observed is statistically significant, found in Cases and absent in Controls and Gene Pool[b.]
**230211372**	232144749	Green et al. (2011)	RDC - SABP + DSM-IV – RDC		**T754S**	C>G	Exon11													Identified as a rare-variant in exon 11.
**230211383**	232144760	Green et al. (2011)	DSM-IV BP1		**P758R**	C>G	Exon11													Identified in an individual with Bipolar I disorder with psychotic symptoms, possibly damaging.
230215556	232148933	Hennah et al. (2009)	Interplay SNPs	**rs821633**		T>C		S492535	25/87/108	19/116/102	16/81/94	88/391/407	0.3113636	0.3248945	0.2958115	0.3199774	**rs821633**	A/G	A	Showed evidence for interplay, either increasing or decreasing risk dependent on presence or absence of rs1538979 or rs821577, a protective variant when on its own.
230216466	232149843	Carless et al. (2011)	Influences brain structure	**rs821639**		A>G		S493445	25/83/109	19/113/104	16/80/94	83/387/416	0.3064516	0.3199153	0.2947368	0.3120767	**rs821639**	C/T	T	
230228872	232162249	Osbun et al. (2011)	AgCC		**L792L**	G>A	Exon12													
230228916	232162293	Song et al. (2010)	Bipolar Spectrum Disorder[c] + CTL		**808 frames**	TCATdel	Exon12													
230238997	232172374	Osbun et al. (2011)	AgCC	rs17773715	**IVS12 – 64**	G>A	Intron12	S515976	8/51/135	12/69/144	7/33/124	24/222/531	0.1726804	0.2066667	0.1432927	0.1737452	rs17773715	A/G	G	
**230239137**	232172514	Moens et al. (2011)	SCZ	rs41271517	E834E	G>A	Exon13	S516116	0/0/209	0/0/236	0/0/173	**0/6/793**	0	0	0	0.0037547	rs41271517	A/G	G	Found only in Cases, No obvious functional effect (though genomically conserved).
230241561	232174938	Carless et al. (2011)	Influences brain structure	**rs9661837**													**rs9661837**	A/G	A	
230243610	232176987	Carless et al. (2011)	Influences brain structure	**rs16856322**		C>T	Exon13	S520589	21/84/110	19/101/119	18/81/87	68/341/422	0.2930233	0.290795	0.3145161	0.2870036	**rs16856322**	C/T	C	
230243820	232177197	Carless et al. (2011)	Influences brain structure	**rs17773946**													**rs17773946**	A/G	A	

[a] A different nucleotide change was observed in the study samples – G>A

Abbreviations : BP1 - Bipolar disorder type I, SCZ - Schizophrenia, UP - Unipolar, CTL - Control, LBC - Lothian Birth Cohort, SABP - Schizoaffective Disorder,Bipolar type, DSM-IV – Diagnostic and Statistical Manual, Fourth Edition, AgCC - Agenesis of the corpus callosum

[b] Gene Pool – 10,000 unrelated control alleles (Song et al. 2008)

[c] Bipolar Spectrum Disorder is defined as bipolar disease meeting DSM-IV criteria for bipolar I disorder, bipolar II disorder, schizoaffective disorder, bipolar type, or bipolar disorder.

[d] HapMap Population – CEU, YRI, CHB, JPT

[e] African American Only

Sorted by Study (see above for by Variant)

Author	dbSNP135	AA change	NT change	Position	Diagnosis	SNP	hg18	hg19	Genotype Distribution BP1	Genotype Distribution SCZ	Genotype Distribution rMDD	Genotype Distribution CTL	Minor Allele Frequency BP1	Minor Allele Frequency SCZ	Minor Allele Frequency UP	Minor Allele Frequency CTL	dbSNP	Obs Allele	Ref Allele	Literature Interpretation
Song et al. (2008)		**A13G**	C>G	Exon1	SCZ + CTL		229829274	231762651												
		**G14A**	G>C	Exon1	SCZ		229829277	231762654												Identified in Cases only.
		**R37W**	C>T	Exon2	SCZ	S173215	229896236	231829613	0/0/211	0/0/232	**0/1/179**	0/0/847	0	0	0.0027778	0				Identified in Cases only.
	rs76175896	**A83V**	C>T	Exon2	SCZ + CTL	S173354	229896375	231829752	**0/1/215**	0/0/236	0/0/188	0/0/860	0.0023148	0	0	0				Identified in both Cases and Gene Pool[b.]
		**S90L**	C>T	Exon2	SCZ	S173375	229896396	231829773	0/0/216	0/0/236	0/0/190	**0/1/857**	0	0	0	0.0005828				Identified in Cases only.
		**Q264C**	C>T	Exon2	CTL		229896917	231830294												Identified in both Cases and Gene Pool[b.]
	rs3738401	**Q264R**	A>G	Exon2	SCZ + CTL	S173897	229896918	231830295	28/86/98	24/94/112	21/84/79	94/354/413	0.3349057	0.3086957	0.3423913	0.3147503	rs3738401	A/G	G	Associated with elevating the risk for Schizophrenia.
	rs144959108	**R418H**	G>A	Exon4	SCZ	S229409	229952430	231885807	0/0/215	**0/1/236**	0/0/181	0/0/861	0	0.0021097	0	0				Identified in Cases only.
	rs78792190	**P432L**	C>T	Exon5	SCZ + CTL	S246514	229969535	231902912	0/3/210	0/2/233	0/0/184	0/6/854	0.0070423	0.0042553	0	0.0034884				Identified in both Cases and Gene Pool[b.]
	rs28930675	**T453M**	C>T	Exon5	SCZ + CTL	S246577	229969598	231902975	0/3/210	0/0/236	0/2/182	0/11/842	0.0070423	0	0.0054348	0.0064478	rs28930675	C/T	C	
		**T603I**	C>T	Exon9	SCZ		230020713	231954090												Identified in Cases only.
	rs6675281	**L607F**	C>T	Exon9	SCZ + CTL	S297703	230020724	231954101	4/39/130	8/46/151	3/25/123	19/179/554	0.1358382	0.1512195	0.102649	0.1442819	rs6675281	C/T	C	Previously associated with elevating the risk for Schizophrenia, no evidence found.
	rs821616	**S704C**	A>T	Exon11	SCZ + CTL	S488200	230211221	232144598	15/57/125	18/80/120	12/59/97	66/300/451	0.2208122	0.266055	0.2470238	0.2643819	rs821616	A/T	T	Associated with elevating the risk for Schizophrenia.
	rs115112816	**E751Q**	G>C	Exon11	SCZ + CTL	S488341	230211362	232144739	0/3/207	0/2/228	0/11/169	0/21/843	0.0071429	0.0043478	0.0305556	0.0121528				
Hennah W and	**rs1765778**		A>G		Increases D	S075958	229798979	231732356	51/102/65	46/118/75	41/91/57	171/403/31	0.4678899	0.4393305	0.457672	0.419482	**rs1765778**	A/G	G	Displayed significant association in all four HapMap populations[d], minor allele associated with decreased DISC1 expression.
	**rs1655297**		T>C		Increases D	S077289	229800310	231733687	34/80/56	36/92/80	26/66/67	105/333/31	0.4352941	0.3942308	0.3710692	0.3629679	**rs1655297**	A/G	G	Displayed significant association in three of the four HapMap populations exception being the Japanese population[d], minor allele associated with decreased DISC1 expression.
	**rs1025526**		T>C		Increases D	S094243	229817264	231750641	32/102/87	31/93/113	26/73/92	95/365/428	0.3755656	0.3270042	0.3272251	0.3125	**rs1025526**	A/G	G	Displayed significant association in three of the four HapMap populations exception being the Japanese population[d], minor allele associated with decreased DISC1 expression.
	**rs6541280**		C>G		Increases D	S098481	229821502	231754879	39/93/62	38/91/93	28/63/55	116/355/32	0.4407216	0.3761261	0.4075342	0.366875	**rs6541280**	C/G	G	Displayed significant association in three of the four HapMap populations exception being the Japanese population[d], minor allele associated with decreased DISC1 expression.
	**rs3738398**		C>G		Increases D	S105942	229828963	231762340	30/47/22	35/49/50	20/43/19	99/228/162	0.540404	0.4440299	0.5060976	0.4355828	**rs3738398**	C/G	G	Displayed significant association in three of the four HapMap populations exception being the Japanese population[d], minor allele associated with decreased DISC1 expression.
	**rs823167**				Increases DISC1 expres		229833853	231767230									**rs823167**	C/T	C	Displayed significant association in three of the four HapMap populations exception being the Japanese population[d], minor allele associated with decreased DISC1 expression.
Hennah et al. (2	**rs1538979**		C>T		Interplay SN	S240470	229963491	231896868	1/38/175	4/46/183	6/41/134	25/201/645	0.0934579	0.1158798	0.1464088	0.1440873	**rs1538979**	A/G	G	Significant association observed in Finnish BP1 males, no significant odds ratios in combined cohorts.
	**rs821577**		T>G		Interplay SN	S410659	230133680	232067057	40/110/66	42/114/83	38/93/60	171/413/29	0.4398148	0.4142259	0.4424084	0.4289773	**rs821577**	G/T	T	Significant association observed in the London BP females, showed evidence for increased risk within the combined cohorts.
	**rs821633**		T>C		Interplay SN	S492535	230215556	232148933	25/87/108	19/116/102	16/81/94	88/391/407	0.3113636	0.3248945	0.2958115	0.3199774	**rs821633**	A/G	A	Showed evidence for interplay, either increasing or decreasing risk dependent on presence or absence of rs1538979 or rs821577, a protective variant when on its own.
Song et al. (2010)		**A13G**	C>G	Exon1	Bipolar Spectrum Disor		229829274	231762651												Occurred in both Cases and Control, not associated with bipolar spectrum disorder.
		**IVS1 + 3**	A>T	Intron1	Bipolar Spectrum Disor		229829306	231762683												Ultra-rare variant observed is statistically significant, found in Cases and absent in Controls and Gene Pool[b.]
	rs143922209	**R25Q**	G>A	Exon2	Bipolar Spe	S173180	229896201	231829578	0/2/213	0/1/235	0/1/188	0/2/871	0.0046512	0.0021186	0.0026455	0.0011455				
		**S209R**	C>G[a]	Exon2	Bipolar Spectrum Disor		229896754	231830131												Ultra-rare variant observed is statistically significant, found in Cases and absent in Controls and Gene Pool[b.]
		**Q264C**	C>T	Exon2	CTL		229896917	231830294												
	rs3738401	**Q264R**	A>G	Exon2	Bipolar Spe	S173897	229896918	231830295	28/86/98	24/94/112	21/84/79	94/354/413	0.3349057	0.3086957	0.3423913	0.3147503	rs3738401	A/G	G	Occurred in both Cases and Control, not associated with bipolar spectrum disorder.
	rs146244276	**P287L**	C>T	Exon2	Bipolar Spe	S173966	229896987	231830364	0/1/215	0/0/237	0/0/188	0/1/864	0.0023148	0	0	0.000578				Occurred in both Cases and Control, not associated with bipolar spectrum disorder.
		**D296G**	A>G	Exon2	CTL		229897014	231830391												
		**R338Q**	G>A	Exon2	Bipolar Spectrum Disor		229897140	231830517												Ultra-rare variant observed is statistically significant, found in Cases and absent in Controls and Gene Pool[b.]
	rs144959108	**R418H**	G>A	Exon4	Bipolar Spe	S229409	229952430	231885807	0/0/215	**0/1/236**	0/0/181	0/0/861	0	0.0021097	0	0				Identified as ultra-rare variant in SCZ study too (Song et al., 2008).
	rs78792190	**P432L**	C>T	Exon5	Bipolar Spe	S246514	229969535	231902912	0/3/210	0/2/233	0/0/184	0/6/854	0.0070423	0.0042553	0	0.0034884				Occurred in both Cases and Control, not associated with bipolar spectrum disorder.
	rs28930675	**T453M**	C>T	Exon5	Bipolar Spe	S246577	229969598	231902975	0/3/210	0/0/236	0/2/182	0/11/842	0.0070423	0	0.0054348	0.0064478	rs28930675	C/T	C	Occurred in both Cases and Control, not associated with bipolar spectrum disorder.
	rs143165003	**P540Q**	C>A	Exon6	Bipolar Spectrum Disor		229973424	231906801												Occurred in both Cases and Control, not associated with bipolar spectrum disorder.
		**S567N**	G>A	Exon8	Bipolar Spectrum Disor		230002487	231935864												
	rs6675281	**L607F**	C>T	Exon9	Bipolar Spe	S297703	230020724	231954101	4/39/130	8/46/151	3/25/123	19/179/554	0.1358382	0.1512195	0.102649	0.1442819	rs6675281	C/T	C	Occurred in both Cases and Control, not associated with bipolar spectrum disorder.
	rs821616	**C704S**	A>T	Exon11	Bipolar Spe	S488200	230211221	232144598	15/57/125	18/80/120	12/59/97	66/300/451	0.2208122	0.266055	0.2470238	0.2643819	rs821616	A/T	T	Occurred in both Cases and Control, not associated with bipolar spectrum disorder.
	rs115112816	**E751Q**	G>C	Exon11	Bipolar Spe	S488341	230211362	232144739	0/3/207	0/2/228	0/11/169	0/21/843	0.0071429	0.0043478	0.0305556	0.0121528				Occurred in both Cases and Control, not associated with bipolar spectrum disorder.
		**T754S**	C>G	Exon11	Bipolar Spectrum Disor		230211372	232144749												Ultra-rare variant observed is statistically significant, found in Cases and absent in Controls and Gene Pool[b.]
		**808 frameshift**	TCATdel	Exon12	Bipolar Spectrum Disor		230228916	232162293												
Carless et al. (2	**rs16854756**				Influences brain structur		229879331	231812708									**rs16854756**	C/T	T	
	**rs3738401**	Q264R	G>A	Exon2	Influences b	S173897	229896918	231830295	28/86/98	24/94/112	21/84/79	94/354/413	0.3349057	0.3086957	0.3423913	0.3147503	**rs3738401**	A/G	G	Observed moderate evidence for cis-regulation in significantly larger population size.
	**rs2487453**		C>T	Intron3	Influences b	S206806	229929827	231863204	35/87/87	30/102/101	26/88/68	115/381/37	0.3755981	0.3476395	0.3846154	0.350344	**rs2487453**	A/G	A	Found to be cis-acting and influences brain structure, specifically thickness of the cingulate cortex
	**rs2793094**				Spatial working memory		229943605	231876982									**rs2793094**	C/T	C	Associations were detected with measures of spatial working memory
	**rs16854954**		T>C		Influences b	S256194	229979215	231912592	24/72/112	25/90/112	16/76/93	71/342/423	0.2884615	0.30837	0.2918919	0.2894737	**rs16854954**	C/T	T	
	**rs2356606**				Influences brain structur		230081663	232015040									**rs2356606**	C/T	T	Found to be associated with thickness of the entorhinal cortex and postcentral gyrus
	**rs9431714**		G>A		Influences b	S364850	230087871	232021248	33/103/76	34/109/92	29/76/79	119/399/36	0.3985849	0.3765957	0.3641304	0.361521	**rs9431714**	A/G	G	
	**rs4658966**		T>C		Major depre	S422216	230145237	232078614	4/49/140	5/52/166	2/38/130	16/179/614	0.1476684	0.1390135	0.1235294	0.1304079	**rs4658966**	C/T	T	Associations were detected with recurrent major depression disorder
	**rs12137417**				Major depressive disord		230152776	232086153									**rs12137417**	A/G	G	Associations were detected with recurrent major depression disorder and lifetime measures of panic
	**rs821639**		A>G		Influences b	S493445	230216466	232149843	25/83/109	19/113/104	16/80/94	83/387/416	0.3064516	0.3199153	0.2947368	0.3120767	**rs821639**	C/T	T	
	**rs9661837**				Influences brain structur		230241561	232174938									**rs9661837**	A/G	A	
	**rs16856322**		C>T	Exon13	Influences b	S520589	230243610	232176987	21/84/110	19/101/119	18/81/87	68/341/422	0.2930233	0.290795	0.3145161	0.2870036	**rs16856322**	C/T	C	
	**rs17773946**				Influences brain structur		230243820	232177197									**rs17773946**	A/G	A	
Green et al. (2011)		**R720R**	G>A	Exon11	RDC – SABP		**230211271**	232144648												Identified in Cases only.
		**L736L**	C>T	Exon11	RDC – SABP		**230211319**	232144696												Identified in Cases only.
	rs115112816	**E751Q**	G>C	Exon11	RDC - SABI	S488341	**230211362**	232144739	0/3/207	0/2/228	0/11/169	0/21/843	0.0071429	0.0043478	0.0305556	0.0121528				Identified in both Cases and Controls.
	rs183265932	**E751A**	A>C	Exon11	RDC – SABP + DSM-IV		**230211363**	232144740												Identified in both Cases and Controls.
		**T754S**	C>G	Exon11	RDC - SABP + DSM-IV		**230211372**	232144749												Identified as a rare-variant in exon 11.
		**P758R**	C>G	Exon11	DSM-IV BP1		**230211383**	232144760												Identified in an individual with Bipolar I disorder with psychotic symptoms, possibly damaging.
Osbun et al. (2011)		**V71L**	G>T	Exon2	AgCC		229896338	231829715												
	rs76175896	**A83V**	C>T	Exon2	AgCC	S173354	229896375	231829752	**0/1/215**	0/0/236	0/0/188	0/0/860	0.0023148	0	0	0				
	rs3738401	**R264Q**	G>A	Exon2	AgCC + CT	S173897	229896918	231830295	28/86/98	24/94/112	21/84/79	94/354/413	0.3349057	0.3086957	0.3423913	0.3147503	rs3738401	A/G	G	Known to be associated with higher risk of developing SCZ, did not reach the statistical significance in this study.
	rs146244276	**P287L**	C>T	Exon2	AgCC + CT	S173966	229896987	231830364	0/1/215	0/0/237	0/0/188	0/1/864	0.0023148	0	0	0.000578				Detected at low frequencies in both Cases and Control.
		**IVS4 – 36**	T>C	Intron4	AgCC		229952259	231885636												
	rs28930675	**T453M**	C>T	Exon5	AgCC + CT	S246577	229969598	231902975	0/3/210	0/0/236	0/2/182	0/11/842	0.0070423	0	0.0054348	0.0064478	rs28930675	C/T	C	Detected at low frequencies in both Cases and Control.
	rs3738402	**L465L**	C>T	Exon5	AgCC	S246612	229969633	231903010	0/14/199	0/9/227	0/13/171	2/43/816	0.0328638	0.0190678	0.0353261	0.0272938	rs3738402	C/T	C	
	rs2492367	**I469I**	C>T	Exon6	AgCC	S250191	229973212	231906589	3/40/176	2/53/185	0/41/151	15/195/672	0.1050228	0.11875	0.1067708	0.127551	rs2492367	C/T	C	
	rs143165003	**P540Q**	C>A	Exon6	AgCC		229973424	231906801												Identified in Cases only.
	rs16854940	**IVS6 + 31**	C>T	Intron6	AgCC	S250449	229973470	231906847	0/14/207	0/9/231	0/13/179	2/45/838	0.0316742	0.01875	0.0338542	0.0276836	rs16854940	C/T	C	
	rs41271515	**IVS6 – 89**	G>A	Intron6	AgCC	S274501	229997522	231930899	0/1/219	0/0/240	0/0/191	0/3/879	0.0022727	0	0	0.0017007	rs41271515	A/G	G	

		**IVS7 + 6**	T>C	Intron7	AgCC		229997670	231931047												
	rs2759354	**IVS8 + 60**	G>A	Intron8	AgCC	S279618	230002639	231936016	0/2/207	0/2/233	0/3/183	0/13/867	0.0047847	0.0042553	0.0080645	0.0073864	rs2759354	C/T	T	
	rs6675281	**L607F**	C>T	Exon9	AgCC + CTl	S297703	230020724	231954101	4/39/130	8/46/151	3/25/123	19/179/554	0.1358382	0.1512195	0.102649	0.1442819	rs6675281	C/T	C	Known to be associated with higher risk of developing SCZ, did not reach the statistical significance in this study.
	rs12133766	**L621L**	G>A	Exon9	AgCC	S297747	230020768	231954145	0/14/173	0/19/200	1/20/137	3/78/682	0.0374332	0.043379	0.0696203	0.0550459	rs12133766	A/G	G	
	rs2794273	**IVS10 + 95**	G>A	Intron10	AgCC	S438331	230161352	232094729	9/61/103	14/78/105	11/55/80	45/253/341	0.2283237	0.2690355	0.2636986	0.2683881	rs2794273	C/T	C	
		**IVS10 – 2**	A>G	Intron10	AgCC		230211153	232144530												Identified in Cases only.
	rs821616	**S704C**	A>T	Exon11	AgCC + CTl	S488200	230211221	232144598	15/57/125	18/80/120	12/59/97	66/300/451	0.2208122	0.266055	0.2470238	0.2643819	rs821616	A/T	T	Known to be associated with higher risk of developing SCZ, did not reach the statistical significance in this study.
		**L792L**	G>A	Exon12	AgCC		230228872	232162249												
	rs17773715	**IVS12 – 64**	G>A	Intron12	AgCC	S515976	230238997	232172374	8/51/135	12/69/144	7/33/124	24/222/531	0.1726804	0.2066667	0.1432927	0.1737452	rs17773715	A/G	G	
Moens et al. (20	rs3738399	7G>A	G>A	5'UTR	SCZ + CTL	S106209	**229829230**	231762607	1/1/65	0/0/99	0/0/68	2/1/414	0.0223881	0	0	0.0059952	rs3738399	A/G	G	Found in both Cases and Controls.
	rs76175896	A83V	C>T	Exon2	SCZ + CTL	S173354	**229896375**	231829752	**0/1/215**	0/0/236	0/0/188	0/0/860	0.0023148	0	0	0	rs76175896			Found in both Cases and Controls, Possible splicing effect.
	rs143796295	W160L	G>T	Exon2	SCZ + CTL	S173585	**229896606**	231829983	0/0/214	**0/1/235**	0/0/187	0/0/858	0	0.0021186	0	0				Found in both Cases and Controls, No obvious functional effect.
	rs3738401	R264Q	G>A	Exon2	SCZ + CTL	S173897	**229896918**	231830295	28/86/98	24/94/112	21/84/79	94/354/413	0.3349057	0.3086957	0.3423913	0.3147503	rs3738401	A/G	G	Found in both Cases and Controls, Possible splicing effect.
	rs3738402	L465L	C>T	Exon5	SCZ + CTL	S246612	**229969633**	231903010	0/14/199	0/9/227	0/13/171	2/43/816	0.0328638	0.0190678	0.0353261	0.0272938	rs3738402	C/T	C	Found in both Cases and Controls, Possible amino acid effect.
	rs2492367	I469I	C>T	Exon6	SCZ + CTL	S250191	**229973212**	231906589	3/40/176	2/53/185	0/41/151	15/195/672	0.1050228	0.11875	0.1067708	0.127551	rs2492367	C/T	C	Found in both Cases and Controls, Possible splicing effect.
	rs2273890	169488T>C	T>C	Splice/Intron7	SCZ + CTL	S274650	**229997671**	231931048	4/60/157	9/56/175	4/44/143	20/213/655	0.1538462	0.1541667	0.1361257	0.142455	rs2273890	A/G	A	Found in both Cases and Controls, Possible splicing effect (5' splice site).
	rs6675281	L607F	C>T	Exon9	SCZ + CTL	S297703	**230020724**	231954101	4/39/130	8/46/151	3/25/123	19/179/554	0.1358382	0.1512195	0.102649	0.1442819	rs6675281	C/T	C	
	rs12133766	L621L	G>A	Exon9	SCZ + CTL	S297747	**230020768**	231954145	0/14/173	0/19/200	1/20/137	3/78/682	0.0374332	0.043379	0.0696203	0.0550459	rs12133766	A/G	G	Found in both Cases and Controls, No obvious functional effect (though genomically conserved).
	rs3082	2105A>G	A>G	3'UTR/Intron9	SCZ + CTL		**230069015**	232002392									rs3082	C/T	T	
	rs821616	S704C	A>T	Exon11	SCZ + CTL	S488200	**230211221**	232144598	15/57/125	18/80/120	12/59/97	66/300/451	0.2208122	0.266055	0.2470238	0.2643819	rs821616	A/T	T	Found in both Cases and Controls, Possible amino acid effect.
	rs115112816	E751Q	G>C	Exon11	SCZ + CTL	S488341	**230211362**	232144739	0/3/207	0/2/228	0/11/169	0/21/843	0.0071429	0.0043478	0.0305556	0.0121528				Found in both Cases and Controls, No obvious functional effect (though genomically conserved).
	rs41271517	E834E	G>A	Exon13	SCZ	S516116	**230239137**	232172514	0/0/209	0/0/236	0/0/173	**0/6/793**	0	0	0	0.0037547	rs41271517	A/G	G	Found only in Cases, No obvious functional effect (though genomically conserved).
Crowley et al. (2				5 upstream	SCZ + CTL	S106075	**229829096**	231762473	0/1/88	0/2/123	0/1/81	0/4/484	0.005618	0.008	0.006098	0.0040984				
	rs143922209	R25Q	G>A	Exon2	SCZ + CTL	S173180	**229896201**	231829578	0/2/213	0/1/235	0/1/188	0/2/871	0.0046512	0.0021186	0.0026455	0.0011455				3' UTR
					SCZ + CTL[e]		**229896323**	231829700												Mis-sense
					SCZ		**229896338**	231829715												Mis-sense, PolyPhen - benign, SIFT - tolerated
		S90L	C>T	Exon2	SCZ	S173375	**229896396**	231829773	0/0/216	0/0/236	0/0/190	**0/1/857**	0	0	0	0.0005828				Mis-sense, PolyPhen - benign, SIFT - tolerated
	rs143796295	W160L	G>T	Exon2	SCZ + CTL	S173585	**229896606**	231829983	0/0/214	**0/1/235**	0/0/187	0/0/858	0	0.0021186	0	0				Mis-sense, PolyPhen - possibly damaging, SIFT - tolerated
	**rs3738401**	Q264R	A>G	Exon2	SCZ + CTL	S173897	229896918	231830295	28/86/98	24/94/112	21/84/79	94/354/413	0.3349057	0.3086957	0.3423913	0.3147503	rs3738401	A/G	G	Mis-sense, PolyPhen - benign, SIFT - tolerated
					SCZ		**229896948**	231830325												Mis-sense, PolyPhen - benign, SIFT - tolerated
	rs146244276	P287L	C>T	Exon2	SCZ + CTL	S173966	**229896987**	231830364	0/1/215	0/0/237	0/0/188	0/1/864	0.0023148	0	0	0.000578				Mis-sense, PolyPhen - benign, SIFT - tolerated
		W359*	G>A	Intron3	CTL	S201916	**229924937**	231858314	0/0/207	0/0/233	0/0/186	**0/1/756**	0	0	0	0.0006605				Non-sense
				Intron3	CTL	S201965	**229924986**	231858363	0/0/216	0/0/235	0/0/191	**0/1/793**	0	0	0	0.0006297				3' UTR
					CTL		**229925021**	231858398												3' UTR
	rs78792190	P432L	C>T	Exon5	SCZ + CTL	S246514	**229969535**	231902912	0/3/210	0/2/233	0/0/184	0/6/854	0.0070423	0.0042553	0	0.0034884				Mis-sense, PolyPhen - possibly damaging, SIFT - damaging
					SCZ[e]		**229973321**	231906698												Mis-sense, PolyPhen - possibly damaging, SIFT - tolerated
	rs143165003	P540Q	C>A	Exon6	SCZ		**229973424**	231906801												Mis-sense, PolyPhen - possibly damaging, SIFT - damaging
					SCZ + CTL[e]		**229973446**	231906823												Splice-region
					SCZ		**230002543**	231935920												Mis-sense, PolyPhen - possibly damaging, SIFT - tolerated
	**rs6675281**	L607F	C>T	Exon9	SCZ + CTL	S297703	230020724	231954101	4/39/130	8/46/151	3/25/123	19/179/554	0.1358382	0.1512195	0.102649	0.1442819	rs6675281	C/T	C	Mis-sense, PolyPhen - benign, SIFT - damaging
					SCZ + CTL		**230161188**	232094565												Splice-region
					SCZ[e]		**230211174**	232144551												Mis-sense, PolyPhen - benign, SIFT - tolerated
	**rs821616**	S704C	A>T	Exon11	SCZ + CTL	S488200	230211221	232144598	15/57/125	18/80/120	12/59/97	66/300/451	0.2208122	0.266055	0.2470238	0.2643819	rs821616	A/T	T	Mis-sense, PolyPhen - unknown, SIFT - damaging
	rs115112816	E751Q	G>C	Exon11	SCZ + CTL	S488341	**230211362**	232144739	0/3/207	0/2/228	0/11/169	0/21/843	0.0071429	0.0043478	0.0305556	0.0121528				Mis-sense, PolyPhen - unknown, SIFT - damaging

[a] A different nucleotide change was observed in the study samples – G>A

Abbreviations : BP1 - Bipolar disorder type I, SCZ - Schizophrenia, UP - Unipolar, CTL - Control, LBC - Lothian Birth Cohort, SABP - Schizoaffective Disorder,Bipolar type, DSM-IV – Diagnostic and Statistical Manual, Fourth Edition, AgCC - Agenesis of the corpus callosum

[b] Gene Pool – 10,000 unrelated control alleles (Song et al. 2008)

[c] Bipolar Spectrum Disorder is defined as bipolar disease meeting DSM-IV criteria for bipolar I disorder, bipolar II disorder, schizoaffective disorder, bipolar type, or bipolar disorder.

[d] HapMap Population – CEU, YRI, CHB, JPT

[e] African American Only
